# SUMOylation Pattern Predicts Prognosis and Indicates Tumor Microenvironment Infiltration Characterization in Bladder Cancer

**DOI:** 10.3389/fimmu.2022.864156

**Published:** 2022-03-28

**Authors:** Qi-Dong Xia, Jian-Xuan Sun, Yang Xun, Jun Xiao, Chen-Qian Liu, Jin-Zhou Xu, Ye An, Meng-Yao Xu, Zheng Liu, Shao-Gang Wang, Jia Hu

**Affiliations:** Department and Institute of Urology, Tongji Hospital, Tongji Medical College, Huazhong University of Science and Technology, Wuhan, China

**Keywords:** SUMOylation, tumor microenvironment, tumor mutation burden, bladder cancer, immunotherapy

## Abstract

**Background:**

SUMOylation is an important component of post-translational protein modifications (PTMs), and bladder cancer (BCa) is the ninth most common cancer around the world. But the comprehensive role of SUMOylation in shaping tumor microenvironment (TME) and influencing tumor clinicopathological features and also the prognosis of patients remains unclear.

**Methods:**

Using the data downloaded from The Cancer Genome Atlas (TCGA) and the Gene Expression Omnibus (GEO), we comprehensively evaluated the SUMOylation patterns of 570 bladder cancer samples, and systematically correlated these SUMOylation patterns with TME immune cell infiltrating characteristics. The SUMO score was constructed to quantify SUMOylation patterns of individuals using principal component analysis (PCA) algorithms.

**Results:**

Two distinct SUMOylation patterns and gene clusters were finally determined. Significant differences in the prognosis of patients were found among two different SUMOylation patterns and gene clusters, so were in the mRNA transcriptome and the landscape of TME immune cell infiltration. We also established a set of scoring system named SUMO score to quantify the SUMOylation pattern of individuals with BCa, which was discovered to be tightly connected with tumor clinicopathological characteristics and could predict the prognosis of patients with BCa. Moreover, SUMO score was a considerable predictive indicator for the survival outcome independent of tumor mutation burden (TMB) and low SUMO score was related to better response to immunotherapy using PD-1 blockade. We also found that there existed a significant relationship between sensitivity to commonly used chemotherapy drugs and SUMO score. Finally, a nomograph based on five features, namely, SUMO score, age, gender, T category, and M category was constructed to predict the survival probability of patients with BCa in 1, 3, and 5 years, respectively.

**Conclusions:**

Our work demonstrated and overviewed the complicated regulation mechanisms of SUMOylation in bladder cancer, and better understanding and evaluating SUMOylation patterns could be helpful in guiding clinical therapeutic strategy and improving the prognosis of patients with BCa.

## Introduction

Post-translational protein modification (PTM) is an important regulatory mechanism which regulates the interaction between protein and other molecules and changes the hydrophobicity, charge state, conformation and stability of proteins, thus affecting their functions in various biological processes (1). To date, more than 450 PTMs have been discovered, such as, acetylation, ubiquitination, phosphorylation, and methylation (2). SUMOylation, a specific kind of post-translational modification with the small ubiquitin-related modifier (SUMO), was identified in the mid-1990s (3) and found to play important roles in cell growth and migration, transcriptional regulation, immune response and tumorigenesis (4).

SUMOylation is a reversible and dynamic process mediated by several conserved proteins, namely, SUMO proteins (SUMO-1, SUMO-2, SUMO-3, and SUMO-4), SUMO E1 enzyme (SAE1-SAE2 heterodimer), SUMO E2 Enzyme (Ubc9), SUMO E3 Ligases, and SUMO Proteases (SENPs). A SUMOylation cycle consists of maturation, activation, conjugation, ligation, and de-modification as described below. Before being conjugated to target protein, SUMO protein should first be activated and matured by proteolytically cleaving the four amino acids at the C terminal to expose the glycine–glycine (GG) motif with the help of SUMO-specific proteases (SUPs), a family member of SENPs. Then the mature SUMO protein is activated by SUMO E1 enzyme and linked to the SAE2 subunit of SUMO E1 enzyme through the formation of a thioester bond in an ATP-dependent manner. Next the activated SUMO protein is transferred to Ubc9 by forming another thioester bond. Finally, with the assistance of a specific SUMO E3 ligase, Ubc9 catalyzes formation of an isopeptide bond between the C-terminal glycine of SUMO protein and a lysine (K) residue in the substrate. SUMOylation is a reversible process since the isopeptide can be hydrolyzed by SENPs, thus releasing SUMO protein to enter the next SUMOylation cycle (2, 5, 6).

Bladder cancer (BCa) is the ninth most common cancer around the world and the fourth most common cancer among men, with an estimated 550,000 new cases and approximately 200,000 related deaths in 2018 (7, 8). It is also considered as the costliest highly prevalent cancer in the USA (9). Advanced age, male, tobacco smoking, exposure to aromatic amines and other chemicals are the main risk factors for the incidence of bladder cancer (8). Bladder cancer can be divided into non-muscle invasive bladder cancer (NMIBC) and muscle invasive bladder cancer (MIBC) according to the depth of tumor infiltration. NMIBC is confined to the mucosa (stage Ta, CIS) or submucosa (stage T1), for which transurethral resection of the bladder (TURB) and adjuvant intravesical chemotherapy instillations with bacillus Calmette–Guerin (BCG) or other drugs after TURB are conventional treatments (10). As for MIBC, which invades the muscle layer of bladder, is usually treated by radical cystectomy (RC) and some pre- or post- adjuvant therapy such as radiotherapy, chemotherapy and immunotherapy according to risk classification (11). The traditional chemotherapy follows a regimen of methotrexate, vinblastine, doxorubicin, and cisplatin (MVAC), which was proved to improve the prognosis of patients who accepted RC, however, this chemotherapy regimen has been replaced by gemcitabine plus cisplatin (GC) as its similar efficacy and better tolerability (12). Nowadays, due to deep studying and enhanced understanding of the tumor microenvironment (TME) and molecular profiling of bladder cancer, immunotherapies targeted at programmed cell death protein 1 (PD-1) and its ligand programmed cell death protein ligand 1 (PD−L1) have been developed over the past several years and expected to improve the survival outcome of patients with BCa in the future (12). However, only approximately 20% of patients have an objective response to immune checkpoint blockade (ICB), therefore novel therapeutic strategies for BCa need to be developed urgently.

SUMOylation has been proved to play critical roles in cell cycle progression by regulating the activity of cyclin-dependent kinases (CDKs), so aberrant SUMOylation can influence cell proliferation and lead to tumorigenesis (13). Many proteins involved in SUMOylation were found to be highly expressed in cancer tissues, namely, ovarian, colon and prostate cancer (14, 15), suggesting SUMOylation was more activated in tumor and tightly linked to tumor growth. SUMOylation can also promote tumorigenesis *via* regulating oncogenes and tumor suppressors like c-Myc and FoxM1. Besides, like other PTMs, SUMOylation can occur in many tumor-associated proteins, thus producing new tumor antigens and providing novel therapeutic targets (16). Many previous studies have investigated the relationship between SUMOylation and BCa. SENP2 was reported to be downregulated in BCa and it could suppress migration and invasion of BCa cell *in vitro* through inhibiting the SUMOylation of transducin β-like 1 and transducin β-like-related 1 (TBL1/TBLR1), thus inhibiting the nuclear translocation of β-catenin and the transcription of matrix metallopeptidase 13 (MMP13) (17). SENP2 was also found to regulate TGF-β signaling *via* the deSUMOylation of TGFβ receptor I (TGF-βRI) in BCa, thus suppressing epithelial–mesenchymal transition (EMT) *in vivo* and *in vitro* (18). Furthermore, Chen et al. also discovered the unique role of SUMOylation in inducing lymph angiogenesis and promoting tumor lymph node (LN) metastasis in BCa. The long noncoding RNA (lncRNA) LN-associated transcript 1 (ELNAT1) could induce Ubc9 overexpression, which would then mediate the SUMOylation of hnRNPA1 and promote packaging of ELNAT1 into the extracellular vesicles (EVs) secreted by bladder cancer cell. Finally, ELNAT1 was transported to human lymphatic endothelial cells (HLECs) by EVs and activated SOX18 transcription to trigger lymph angiogenesis (19). Given its specific roles in promoting tumorigenesis, metastasis and progression in BCa, SUMOylation could become a latent target for precise treatment in the future.

However, all the above studies focused on only one or two SUMOylation related proteins but no previous studies have investigated and summarized the comprehensive effect of SUMOylation. So, here in this article, we divided the patients with BCa into two SUMOylation patterns according to the expression levels of SUMOylation related genes and performed survival analyses, then we further explored the hallmark pathways and TME immune cell infiltration characteristics in distinct SUMOylation patterns and surprisingly found SUMOylation patterns were significantly connected with TME. Next, we discovered 1,934 SUMOylation cluster related differentially expressed genes (DEGs), classified the patients into two distinct genomic subgroups and explored the interaction between SUMOylation patterns and gene patterns. Moreover, we established a set of scoring system to quantify the SUMOylation pattern in individuals and explored the characteristics of SUMOylation in tumor clinicopathological characteristics, tumor somatic mutation, immunotherapy, and chemotherapy. Finally, we constructed a nomograph based on five features, namely, SUMO score, age, gender, T category, and M category to predict the survival probability of patients with BCa in 1, 3, and 5 years, respectively.

## Materials and Methods

### Data Sources

The key words “SUMOylation” was used to search for related reactome gene sets in the Molecular Signatures Database (MSigDB, https://www.gsea-msigdb.org/gsea/msigdb). The transcriptional profiles and corresponding clinical information of bladder datasets GSE13507 and TCGA_BLCA were separately downloaded from the Gene Expression Omnibus database (GEO, https://www.ncbi.nlm.nih.gov/gds) and the Cancer Genome Atlas Program (TCGA, https://portal.gdc.cancer.gov/). Transcriptional data and corresponding clinical information of immunotherapy cohort IMvigor-210 were obtained from R package “IMvigor210CoreBiologies” (20, 21). The predicted response to checkpoint blockade of TCGA_BLCA datasets by TCIA method was retrieved from the Cancer Immunome Atlas (TCIA, https://tcia.at/home) (22).

### Enhanced SUMOylation Related Pathway and SUMOylation Patterns in Bladder Cancer

Having obtained 6 SUMOylation related gene sets and TCGA_BLCA dataset, we conducted gene sets enrichment analysis (GSEA) between bladder cancer tissues and normal tissues to investigate whether there existed enhanced or weakened SUMOylation functions in bladder cancer compared to the normal samples (23). Then we merged the transcriptional matrix of bladder cancer samples in TCGA_BLCA and GSE13507 dataset, and eliminated the batch effects by the “combat” algorithm using R package “sva”. Following this, we extract the expression values of all genes enrolled in these 6 sumoylation related gene sets for all bladder cancer samples. Non-negative matrix factorization (NMF) algorithm was performed to identify the characteristics of SUMOylation patterns. Then we separately investigated the differential overall survival (Kaplan–Meier method survival curve and log-rank test), enhanced sumoylation related functions [gene set variation analysis (GSVA)], and immune infiltrations [single sample gene set enrichment analysis (ssGSEA)] between SUMOylation patterns. Besides, the principal components analysis (PCA) was carried out to check the discrimination of the SUMOylation patterns.

### Differentially Expressed Genes Between SUMOylation Patterns, DEGs Based Consensus Cluster, and Establishment of the SUMO Score

R package “limma” was used to screen differentially expressed genes (DEGs) defined by the |log_2_ fold change (FC)| >0 and adjusted p-value <0.001, then univariate Cox regression was conducted to check those DEGs with prognostic values. Following this, GO and KEGG gene set enrichment analysis were used to further explore the potential mechanisms or functions influenced by these DEGs with prognostic value. R package “ConsensusClusterPlus” was used to perform unsupervised consensus clustering. Similarly, prognosis differences, differentially enhanced or weakened functions and pathways were compared between DEGs based consensus clusters. Following this, the PCA algorithm was carried out to distinguish the molecular characteristics of these DEGs with prognostic value, and the SUMO score was calculated by the following formula:


SUMO score = Σ(PC1+PC2)

In the above formula. PC1 and PC2 represent two dimension expression patterns of these DEGs with prognostic value in all samples. Then the summary of these two scores can represent the SUMOylation levels of each sample to some extent.

### Verification of the SUMO Score and External Dataset Validation in GSE69795 and GSE70691

Each sample has obtained a corresponding SUMO score according to its transcriptional matrix, then we firstly checked the best cut-off value of SUMO score in the TCGA_BLCA cohort to improve the survival predicting capability and obtain a best area under curve (AUC) by using R package “survminer”. Then all samples from both TCGA_BLCA and GSE13507 cohorts were divided into high-/low-SUMO score groups. Then the survival analysis was performed to verify the efficacy of the SUMO score in the TCGA_BLCA cohort, GSE13507 cohort, and all samples. Besides, we also performed survival analyses using external datasets GSE69795 and GSE70691 for further validation. Also, a gene set variation analysis (GSVA) was conducted to compare the differential active SUMOylation related functions between high SUMO score group and low SUMO score group. Immune cell infiltrations, immune related pathways, and immune related functions were quantified by the single sample gene set enrichment analysis (ssGSEA) algorithm. The correlation between SUMO score and ssGSEA results were conducted by the SPEARMAN correlation test. Having discovered the correlation between SUMO score and immune cell infiltrations, we wondered the correlation between SUMO score and tumor mutation burden (TMB). Thus, TMB of each sample was calculated by its somatic mutation profiles, and we further explored the correlation between SUMO score and TMB, then took these two factors to conduct joint prediction of the overall survival. Finally, the differences in the mutation atlas were compared between high SUMO score group and low SUMO score group by χ^2^ test.

### Prediction of the Response to Chemotherapy/Immunotherapy and External Dataset Validation in IMvigor-210 Cohort

Having checked the effectiveness of the SUMO score, we wondered about the correlation between SUMO score and drug sensitivity to either immunotherapy or chemotherapy. Thus, we conducted three different algorithms to predict the response of each sample to the immunotherapy: TIDE (24), TCIA (22), and submap (25). All the predicted response to immunotherapy was compared by Wilcoxon or χ^2^ test. Besides, we calculated the SUMO score of each patient in IMvigor-210 cohort to externally validate the predicted response to immunotherapy. Responses to anti-PD-L1 immunotherapy in IMvigor-210 cohort were also compared between high- or low-SUMO score groups. As for the drug sensitivity to the chemotherapy, we used the “ProPhetic” package to separately predict the drug sensitivity to two commonly used chemotherapy regimens in bladder cancer (26), namely, GC scheme (gemcitabine plus cisplatin) and MVAC scheme (methotrexate, vinblastine, doxorubicin, and cisplatin). Furthermore, we compared the differential drug sensitivity to chemotherapy between high-/low-SUMO score groups.

### Subgroup Analysis and Construction of SUMO Score Based Nomogram

Detailed clinicopathological characteristics of both TCGA_BLCA cohort and GSE 13507 cohort were collected and sorted. All samples were divided into several sub-groups containing age (>65 or ≤65), gender (male or female), grade (high grade or low grade), T stage (Ta or T1–2 or T3–4), N stage (N0 or N1–3 or Nx), M stage (M0 or M1 or Mx), and survival status (dead or alive), all of which were used to check the efficacy of SUMO score in different subgroups. Following this, we performed both univariate Cox regression and multivariate Cox regression to discover the potential independent prognostic factors between age, gender, grade, T stage, N stage, M stage, and SUMO scores. Finally, we constructed a nomogram including the independent prognostic factors, calculated the corresponding concordance index (C-index), and plotted the calibration curves and receiver operating characteristic (ROC) curve to verify the effectiveness of the nomogram.

### Statistical Analysis

All the data cleaning, statistical analysis, and pictures plotting were conducted by R program version 4.1.1. A p-value <0.05 was defined as the statistically significant.

## Results

### The Landscape of SUMOylation and the Establishment of SUMOylation Patterns in Bladder Cancer

**Figure 1:**
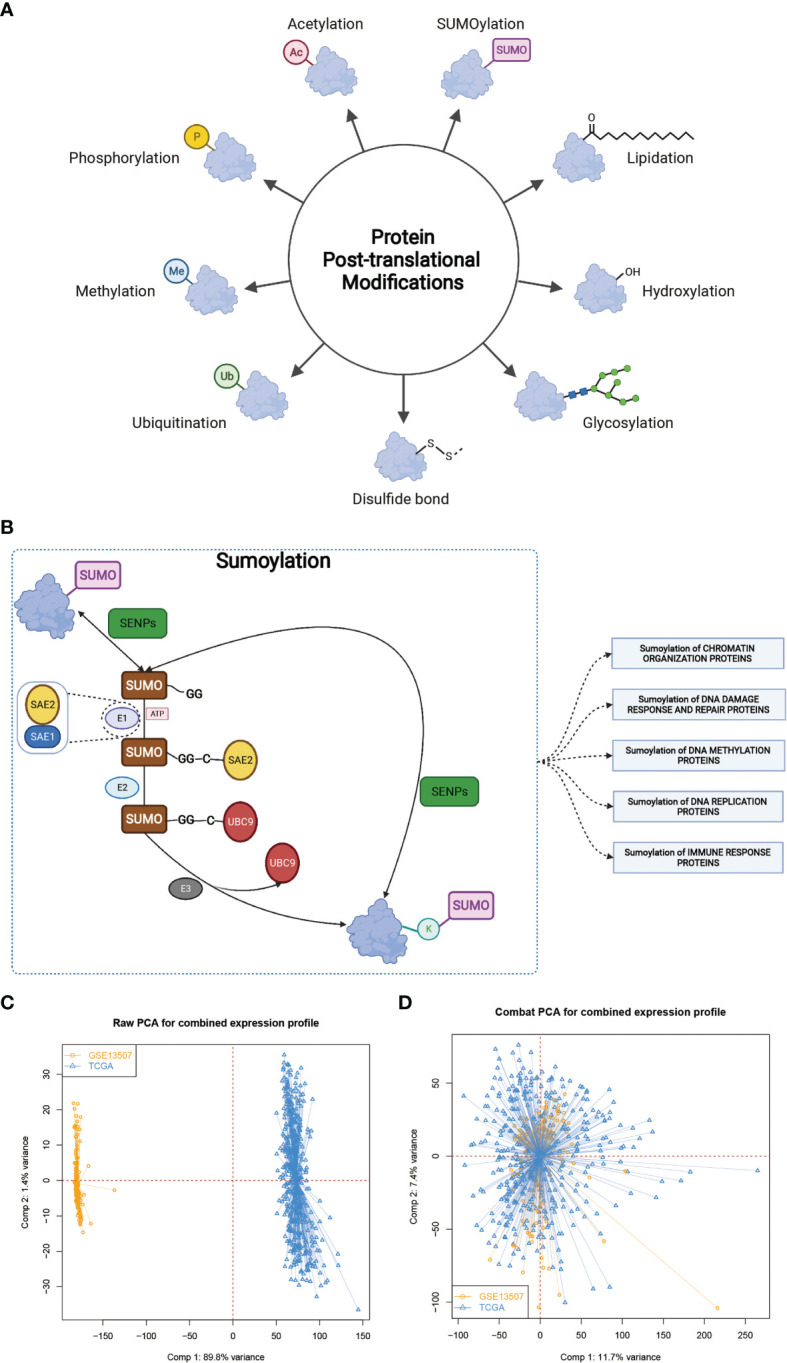
The landscape of SUMOylation and the combination of two different datasets. **(A, B)** Various types of protein post-translational modifications and summary of SUMOylation process, SUMOylation related biological processes, and enzymes involved in these processes. **(C, D)** Principal component analysis for the expression profiles of common genes before and after combination of GSE13507 and TCGA-BLCA cohort. Before processing, two subgroups without intersection were identified, indicating the GSE13507 and TCGA-BLCA samples were well distinguished based on the expression profiles of their common genes, while the two datasets merged together well after processing. Samples from GSE13507 were marked with yellow and samples from TCGA-BLCA marked with blue.

**Figure 2:**
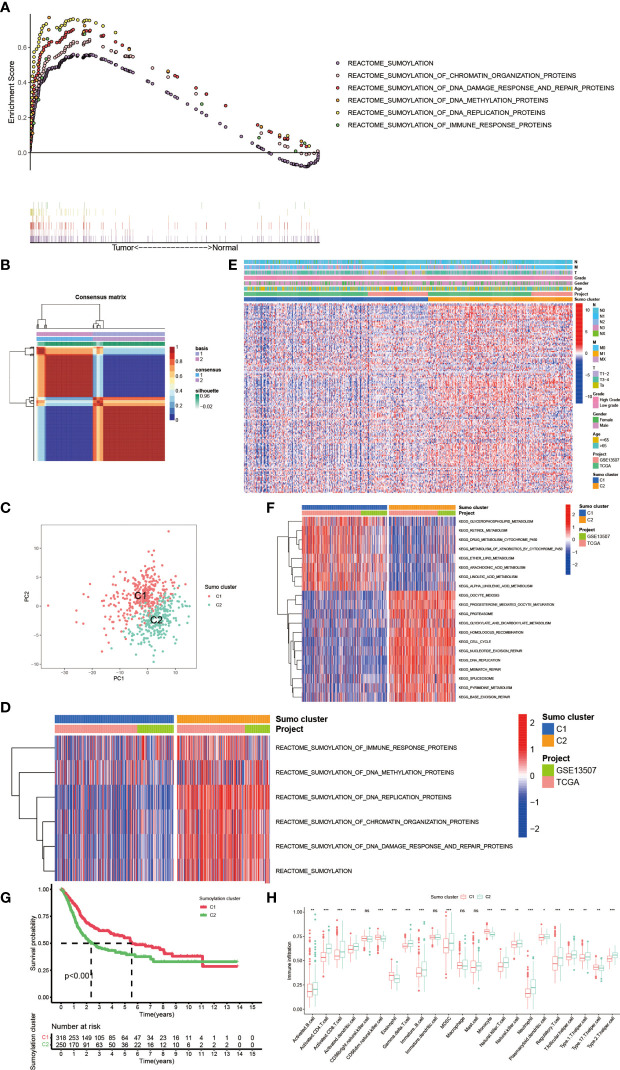
The generation of SUMOylation patterns and clinical and biological characteristics, hallmark pathways, and TME cell infiltration characteristics in distinct SUMOylation patterns. **(A)** The enrichment score of genes involved in SUMOylation and SUMOylation related biological processes between tumor and normal adjacent tumor tissue. **(B)** Connectivity matrix for patients with bladder cancer in the meta-cohort by NMF when k = 2. **(C)** Principal component analysis for the transcriptome profiles of two SUMOylation patterns, showing a remarkable difference on transcriptome between different SUMOylation patterns. **(D)** Transcriptome profiles of genes involved in SUMOylation and SUMOylation related biological processes between the two distinct SUMOylation patterns. **(E)** Heat map for the relationship between clinical characteristics and SUMOylation patterns. **(F)** GSVA enrichment analysis showing the activation states of biological pathways in distinct SUMOylation patterns. The heatmap was used to visualize these biological processes, and red represented activated pathways and blue represented inhibited pathways. The project and Sumo cluster were used as sample annotations. **(G)** Kaplan–Meier curves indicated SUMOylation patterns were markedly related to overall survival of 568 patients in meta-cohort, of which 318 cases were in SUMO cluster C1 and 250 cases in SUMO cluster C2 (P <0.001, Log-rank test). **(H)** The abundance of each TME infiltrating cell in two SUMOylation patterns. The upper and lower ends of the boxes represented interquartile range of values. The lines in the boxes represented median value, and black dots showed outliers. The asterisks represented the statistical p-value (ns, no significance; *P < 0.05; **P < 0.01; ***P < 0.001).

PTM, which includes acetylation, ubiquitination, phosphorylation, methylation, SUMOylation and so on (**Figure 1A**), was found to play vital roles in many biological processes such as substrates transportation (27), signaling transduction (28), autophagy regulation (29), immune responses (30) and many others. As mentioned above, SUMOylation, as an important component of various PTMs, is a reversible process regulated by several highly conserved SUMO family proteins, namely, SUMO-1, SUMO-2, SUMO-3, SUMO-4, SUMO E1-activating enzyme consisting of two subunits named SUMO activating enzyme subunit 1 (SAE1) and SAE2, SUMO−conjugating enzyme E2 (SUMO E2 or Ubc9), SUMO E3 ligase and sentrin/SUMO-specific proteases (SENPs) (2, 5, 6), and mainly participates in nuclear function and intracellular signaling like chromatin organization, DNA damage response and repair, DNA methylation, DNA replication and immune response (**Figure 1B**) (31). Here in **Supplementary Table 1**, we summarized genes involved in protein SUMOylation modification and SUMOylation related biological processes mentioned in **Figure 1B**. One dataset (GSE13507) with complete survival and clinical information was downloaded from the GEO database. Next, we used Combat R packages to eliminate the heterogeneity between the GSE13507 and the TCGA-BLCA cohort and enrolled them into a new meta-cohort. Before processing, we could easily distinguish the two datasets by principal component analysis (PCA) (**Figure 1C**), while the two datasets merged together well after processing (**Figure 1D**). The characteristics of these patients are shown in **Table 1**. As shown in **Figure 2A**, we found significant differences in the activation of SUMOylation related pathways between tumor and normal adjacent tumor tissue, and all these pathways were more activated in tumor than in normal adjacent tumor tissue. To further investigate the interaction between SUMOylation and tumor characteristics, we used Nonnegative Matrix Factorization (NMF) clustering to divide the patients in the meta-cohort into two SUMOylation patterns, termed as SUMO Cluster C1 and SUMO Cluster C2, based on choosing k = 2 as the optimal k value after calculating the cophenetic correlation coefficients of NMF (**Figure 2B** and **Supplementary Figure 1**). Using PCA analysis, we could easily distinguish the two SUMOylation patterns (**Figure 2C**), which revealed a remarkable difference in transcriptional profile of the SUMOylation related genes between the two different SUMOylation patterns. SUMO cluster C2 was characterized by higher expression level of SUMOylation related genes than SUMO cluster C1, indicating enhanced SUMO modification in SUMO cluster C2 (**Figure 2D**). The clinical landscape of the two distinct SUMOylation patterns is shown in **Figure 2E** and there seemed to exist no significant differences in several clinical characteristics such as gender, age and TNM categories between the two SUMOylation patterns. However, a survival analysis for the two SUMO clusters revealed the particularly prominent survival advantage in patients in SUMO cluster C1 (**Figure 2G**).

**Table 1:** Basic characteristics of the included patients.

	Overall	GSE13507	TCGA	p
n	568	165	403	
Status = Alive/Dead (%)	323/245 (56.9/43.1)	96/69 (58.2/41.8)	227/176 (56.3/43.7)	0.755
Age [mean (SD)]	67.22 (11.08)	65.18 (11.97)	68.06 (10.60)	0.005
Gender = Female/Male (%)	135/433 (23.8/76.2)	30/135 (18.2/81.8)	105/298 (26.1/73.9)	0.058
Grade (%)				<0.001
High Grade	440 (77.5)	60 (36.4)	380 (94.3)	
Low Grade	125 (22.0)	105 (63.6)	20 (5.0)	
Unknown	3 (0.5)	0 (0.0)	3 (0.7)	
T (%)				<0.001
T1	83 (14.6)	80 (48.5)	3 (0.7)	
T2	149 (26.2)	31 (18.8)	118 (29.3)	
T3	210 (37.0)	19 (11.5)	191 (47.4)	
T4	69 (12.1)	11 (6.7)	58 (14.4)	
Ta	24 (4.2)	24 (14.5)	0 (0.0)	
Unknown	33 (5.8)	0 (0.0)	33 (8.2)	
M (%)				<0.001
M0	351 (61.8)	158 (95.8)	193 (47.9)	
M1	18 (3.2)	7 (4.2)	11 (2.7)	
MX	197 (34.7)	0 (0.0)	197 (48.9)	
Unknown	2 (0.4)	0 (0.0)	2 (0.5)	
N (%)				<0.001
N0	385 (67.8)	151 (91.5)	234 (58.1)	
N1	54 (9.5)	8 (4.8)	46 (11.4)	
N2	79 (13.9)	4 (2.4)	75 (18.6)	
N3	8 (1.4)	1 (0.6)	7 (1.7)	
Nx	1 (0.2)	1 (0.6)	0 (0.0)	
NX	36 (6.3)	0 (0.0)	36 (8.9)	
Unknown	5 (0.9)	0 (0.0)	5 (1.2)	
Sumo_score (median [IQR])	-0.91 [-17.96, 19.63]	−2.70 [−16.23, 9.56]	0.31 [−20.25, 22.64]	0.575
group = High/Low (%)	274/294 (48.2/51.8)	73/92 (44.2/55.8)	201/202 (49.9/50.1)	0.26

### Hallmark Pathways and TME Cell Infiltration Characteristics in Distinct SUMOylation Patterns

In order to further explore the latent biological behavior differences behind the distinct SUMOylation patterns, we performed gene set variation analysis (GSVA) enrichment analysis based on KEGG datasets. As shown in **Figure 2F**, SUMO cluster C1 was dramatically enriched in pathways associated with metabolism, such as glycerophospholipid metabolism, retinol metabolism, ether lipid metabolism, and drug metabolism mediated by cytochrome P450, while SUMO cluster C2 showed enrichment in pathways related to cell proliferation and tumorigenesis, such as cell cycle, DNA mismatch repair, base excision repair, and DNA replication. Then we analyzed the TME immune cell infiltration and were surprised to find that SUMOylation patterns were closely linked to nearly all kinds of immune cells (**Figure 2H**). SUMO cluster C1 was significantly enriched in few immune cells such as natural killer cell (NK cell), eosinophil, monocyte, and Type 17 T helper cell (Th17), while SUMO cluster C2 exhibited remarkable enrichment in most immune cells such as activated B cell, activated CD4^+^ T cell, activated CD8^+^ T cell, activated dendric cell (DC), myeloid derived suppressor cell (MDSC), natural killer T cell (NKT cell), Type 1 T helper cell (Th1), Type 2 T helper cell (Th2), and regulatory T cell (Treg). However, patients in SUMO cluster C2 did not show a corresponding survival advantage (**Figure 2G**). MDSC is an important component of TME and can perform immune suppressive function *via* various mechanisms, namely, producing nitric oxide (NO), reactive oxygen species (ROS) and mostly anti-inflammatory cytokines such as IL-10 and TGF-β, eliminating key nutrition factors which are vital to T cell proliferation, and preventing CD8^+^ T cell from entering the tumor region (32, 33). Treg is another type of immunosuppressive cell in TME which can interfere the presentation of tumor associated antigens and attenuating cytotoxic T lymphocytes (CTL)-mediated cytotoxicity by influencing the release of cytolytic granules, which was demonstrated to contribute to immune evasion and tumorigenesis (34, 35). As SUMO cluster C2 was remarkably enriched in MDSC and Treg, we speculated these two immunosuppressive cell types could play a crucial role in the poorer clinical outcome of patients with this SUMOylation pattern.

### Generation of SUMOylation Gene Signatures and Functional Annotation

**Figure 3:**
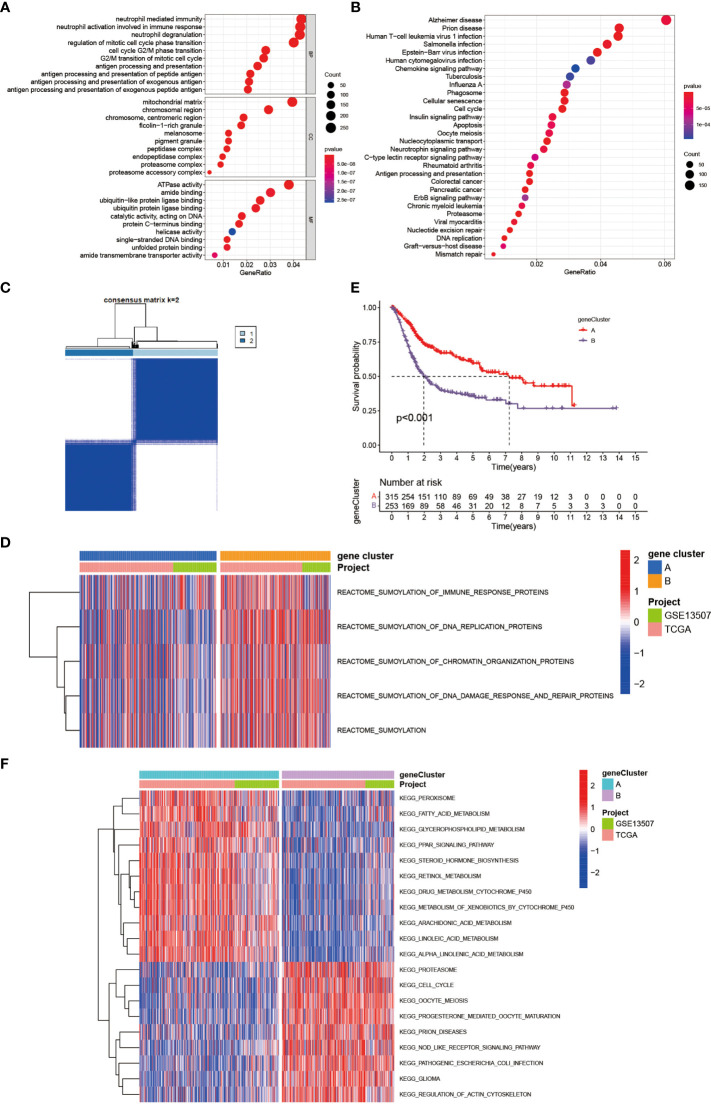
Generation of SUMOylation gene signatures and functional annotation. **(A)** Functional annotation for SUMO cluster related DEGs using GO enrichment analysis. The color depth of the barplots and plots represented the number of genes enriched. The pathways were grouped by cellular component (CC), molecular function (MF) and biological process (BP). **(B)** Functional annotation for SUMO cluster related DEGs using KEGG enrichment analysis. The color depth of the barplots and plots represented the number of genes enriched. **(C)** Unsupervised clustering of 1,934 SUMO cluster related DEGs with prognostic value in meta-cohort and consensus matrices for k = 2. **(D)** Transcriptome profiles of genes involved in SUMOylation and SUMOylation related biological processes between the two distinct gene clusters. **(E)** Kaplan–Meier curves indicated SUMOylation genomic phenotypes were markedly related to overall survival of 568 patients in meta-cohort, of which 315 cases were in gene cluster A and 253 cases in gene cluster B (P <0.001, Log-rank test). **(F)** GSVA enrichment analysis showing the activation states of biological pathways in distinct gene clusters. The heatmap was used to visualize these biological processes, and red represented activated pathways and blue represented inhibited pathways. The project and gene cluster were used as sample annotations.

To further investigate the latent biological behavior of each SUMOylation pattern, we used the limma R package to discover SUMO cluster related DEGs. A total of 6,140 DEGs were identified between the two distinct SUMOylation patterns and finally we screened out 1,934 genes with prognostic value using univariate COX regression for further analysis. Then we performed GO and KEGG enrichment analyses for the DEGs by using the clusterProfiler R package. The results of the GO enrichment analysis showed enrichment in biological process (BP), namely, neutrophil mediated immunity, cell cycle and antigen presentation, cellular component (CC), namely, chromosome and mitochondrial, and molecular function (MF), namely, ubiquitin-like protein ligase binding (**Figure 3A**). The genes in the KEGG analysis also exhibited enrichment in pathways related to immunity, cancer, DNA repair and replication, which was consistent with previous results (**Figure 3B**). The above results demonstrated again that SUMOylation was tightly linked with nuclear function, intracellular signaling and immune response. Then we performed unsupervised clustering analyses based on the 1,934 SUMO cluster related DEGs to dig out the potential regulation mechanism. We chose k = 2 as the optimal k value and successfully classified the patients into two distinct genomic subgroups using the unsupervised clustering algorithm (**Figure 3C** and **Supplementary Figures 2A–H**). Then the cumulative distribution function (CDF) curve and scree plot were used to verify the rationality of the grouping (**Supplementary Figures 2I, J**). The track plot showed the details of grouping (**Supplementary Figure 2K**). We named these patterns as gene cluster A and gene cluster B, respectively. Similarly, gene cluster B was characterized by higher expression level of SUMOylation related genes than gene cluster A, indicating enhanced SUMO modification in gene cluster B (**Figure 3D**). Thus, it was not surprising to find patients in gene cluster A had a better clinical outcome than those in gene cluster B (**Figure 3E**). We also performed GSVA enrichment analysis using KEGG datasets between the two distinct gene clusters (**Figure 3F**). To our surprise, gene cluster A was also remarkably enriched in pathways associated with metabolism, such as glycerophospholipid metabolism, fatty acid metabolism, retinol metabolism, and drug metabolism mediated by cytochrome P450, while gene cluster B exhibited enrichment in pathways linked to cell cycle and immune response to infection, which was approximately in accordance with the results in SUMOylation patterns. Interestingly, we also found peroxisome proliferator-activated receptors (PPAR) signaling was dramatically activated in gene cluster A. Previous studies have indicated that the activation of PPARγ signaling can reduce the level of insulin-like growth factor-1 (IGF-1) in the blood, which was proven to promote tumorigenesis (36), thus interfering the growth of tumor in pancreas, colon, liver, and prostate (37). However, in bladder cancer, studies have shown that PPARγ signaling could enhance the risk of developing bladder cancer (38, 39), and promote the progression and metastasis of bladder cancer *via* providing a tumor-promoting microenvironment (37, 40). But the results of our analyses showed the activation of PPARγ was related to better prognosis in patients with BCa. Overall, the role of PPARγ signaling in bladder cancer is still controversial and further studies are needed in the future.

### Establishment of SUMO Score and Characteristics of SUMOylation in Tumor Mutation Burden and TME Cell Infiltration

**Figure 4:**
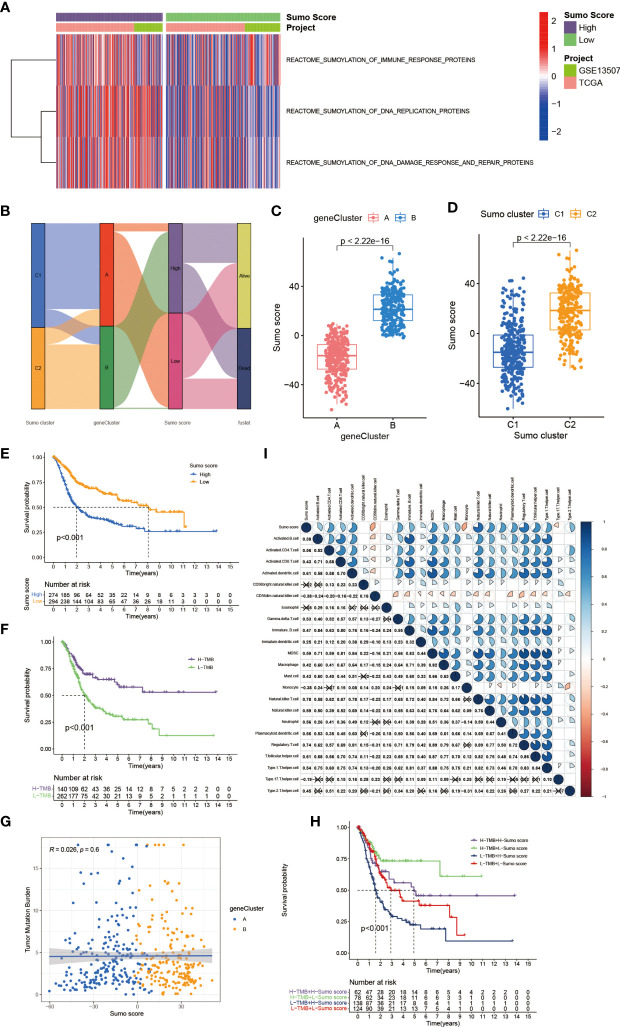
The establishment of SUMO score and its interaction with tumor mutation burden and TME cell infiltration. **(A)** Transcriptome profiles of genes involved in SUMOylation related biological processes between patients with high and low SUMO score. **(B)** Sankey diagram showing the changes of SUMO clusters, gene clusters and SUMO score and final survival status. **(C)** Differences in SUMO score among two gene clusters in meta-cohort. The Wilcoxon test was used to compare the statistical difference between two gene clusters (P <0.001). **(D)** Differences in SUMO score among two SUMO clusters in meta-cohort (P <0.001, Wilcoxon test). **(E)** Survival analyses for low (294 cases) and high (274 cases) SUMO score patient groups in meta-cohort using Kaplan–Meier curves (P <0.001, Log-rank test). **(F)** Survival analyses for patients with low (262 cases) and high (140 cases) tumor mutation burden in the TCGA-BLCA cohort using Kaplan–Meier curves (P <0.001, Log-rank test). **(G)** Linear regression analysis for tumor mutation burden and SUMO score. The dot represented each sample, and the color of the dot represented the gene cluster. Blue, gene cluster A; orange, gene cluster B (R = 0.026, P = 0.6). **(H)** Survival analyses for four groups grouped according to tumor mutation burden and SUMO score in the TCGA-BLCA cohort using Kaplan–Meier curves, namely, 62 cases in high TMB and high SUMO score group, 78 cases in high TMB and low SUMO score group, 138 cases in low TMB and high SUMO score group and 124 cases in low TMB and low SUMO score group. The high TMB and low SUMO score group showed significantly better overall survival than the other three groups (p <0.001, Log-rank test). **(I)** Correlations between SUMO score and the known immune cells in meta-cohort using Spearman analysis. Negative correlation was marked with blue and positive correlation with red. The number represented the correlation coefficient and the cross meant lack of statistical significance.

The above analyses have revealed a landscape of the non-negligible role SUMOylation played in BCa based on the patient population. Given the individual heterogeneity and complexity of SUMOylation, we next explored the latent SUMOylation pattern in individuals. Based on the expression levels of these prognostic DEGs, we constructed a set of scoring system named SUMO score to quantify the SUMOylation pattern of individual patients with BCa. Significant differences were observed in the genes participating in SUMOylation related to immune response, DNA replication, and DNA damage response between patients with high and low SUMO score (**Figure 4A**). Patients with high SUMO score showed higher expression levels in the transcriptional profile of the SUMOylation related genes. The Sankey diagram was used to visualize the attribute changes of individual patients (**Figure 4B**). Not surprisingly, significant differences in SUMO score were also found between different gene cluster (**Figure 4C**) and SUMO cluster (**Figure 4D**). Gene cluster B exhibited higher SUMO score and so did SUMO cluster C2. Moreover, we also observed a significant survival advantage in patients with low SUMO score in all of the TCGA-BLCA cohort (**Supplementary Figure 3A**), GSE13507 cohort (**Supplementary Figure 3B**) and the meta cohort (**Figure 4E**), which was consistent with patients in SUMO cluster C1 (**Figure 2G**) and gene cluster A (**Figure 3E**). We also performed survival analyses in external datasets GSE69795 (**Supplementary Figure 4A**) and GSE70691 (**Supplementary Figure 4B**) for further verification. Although lacking statistical significance, obvious survival differences were observed between patients in high and low SUMO score groups.

**Figure 5:**
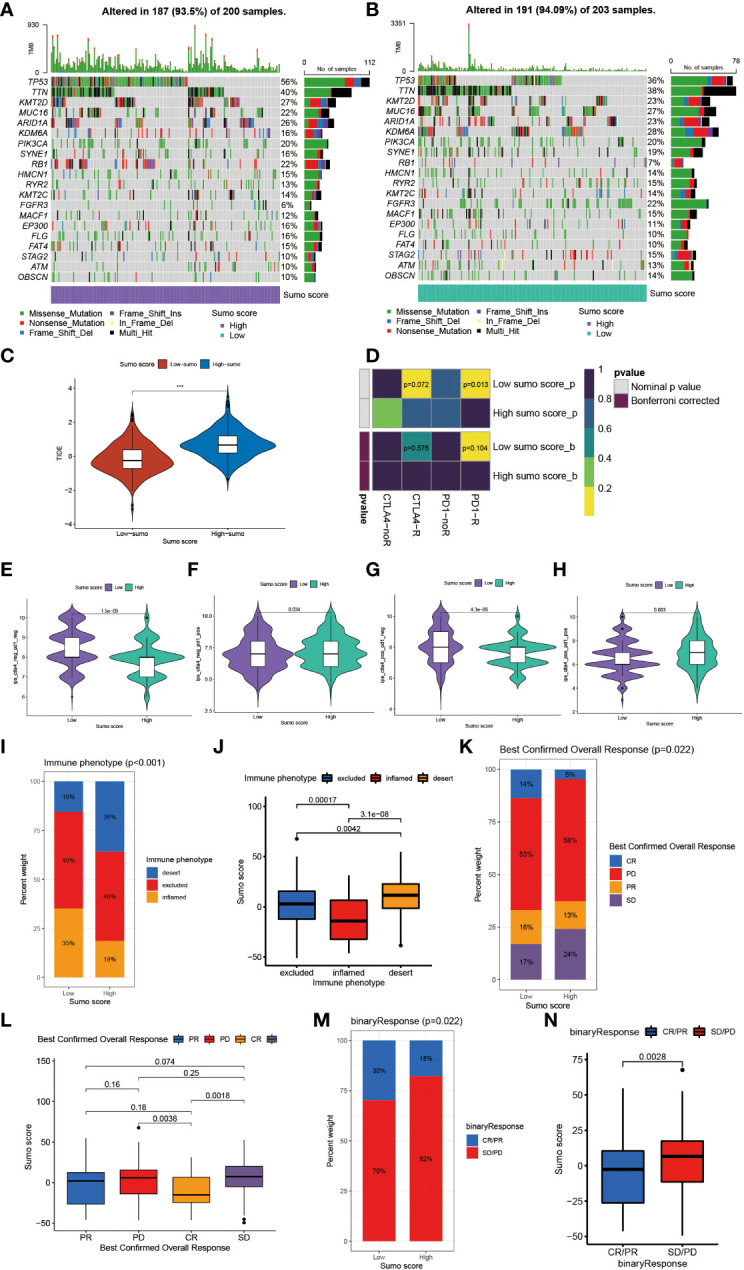
The role of SUMOylation patterns in immunotherapy. **(A, B)** The waterfall plot of tumor somatic mutation established by those with high SUMO score **(A)** and low SUMO score **(B)**. Each column represented individual patients. The upper barplot showed TMB. The number on the right indicated the mutation frequency in each gene. The right barplot showed the proportion of each variant type. **(C)** Differences in the TIDE score between high and low SUMO score groups in meta-cohort (***P <0.001, Wilcoxon test). **(D)** The similarity of gene expression profiles between SUMO score and bladder cancer patients treated with immune checkpoint blockade (ICB). CTLA4-noR, patients no respond to anti-CTLA4 treatment, CTLA4-R, patients respond to anti-CTLA4 treatment, PD1-noR, patients no respond to anti-PD-1 treatment, PD1-R, patients respond to anti-PD-1 treatment. **(E–H)** The violin diagram showed the differences of response index between high and low SUMO score groups among four subgroups. **(E)** If no immunotherapy was conducted, the high SUMO score resulted in a poor prognosis compared to low SUMO score (P <0.001, Wilcoxon test). **(F)** If only anti-PD1 immunotherapy was used, the low SUMO score resulted in a poor prognosis compared to high SUMO score (P = 0.034, Wilcoxon test). **(G)** If only anti-CTLA4 immunotherapy was used, the higher SUMO score group tended to get a poorer therapeutic efficacy compared to low SUMO score group (P <0.001, Wilcoxon test). **(H)** When anti-PD1 and anti-CTLA4 immunotherapy methods were simultaneously adopted, the high SUMO score group might get significantly better prognosis compared to low SUMO score group (P = 0.003, Wilcoxon test). **(I)** The proportion of three immune phenotypes in low or high SUMO score groups. **(J)** Differences in SUMO score among immune excluded, immune inflamed and immune desert phenotypes in IMvigor 210 cohort (Kruskal–Wallis H test). **(K)** The proportion of four different immunotherapy responses in low or high SUMO score groups. CR, complete response; PD, progressive disease; PR, partial response; SD, stable disease. **(L)** Differences in SUMO score among four different immunotherapy responses in IMvigor 210 cohort (Kruskal–Wallis H test). **(M)** The proportion of two binary immunotherapy responses in low or high SUMO score groups. **(N)** Differences in SUMO score among two binary immunotherapy responses in IMvigor 210 cohort (Kruskal–Wallis H test).

Tumor mutation burden (TMB) has been considered a potential biomarker to predict the response to treatment with ICBs. High TMB could predict high ICB efficacy in bladder cancer (41, 42). A multiple cancer research has also revealed patients with higher somatic TMB were associated with better overall survival (43). Consistent with previous studies, we also found patients with high TMB had better clinical outcome compared to those with low TMB (**Figure 4F**). However, we did not find a significant correlation between TMB and SUMO score (**Figure 4G**). Then, we further analyzed the distribution differences of somatic mutation between low and high SUMO score groups in the TCGA-BLCA cohort using maftools R package. As shown in **Figures 5A, B** and **Table 2**, in general, there were no obvious distribution differences of TMB in most mutated genes between low and high SUMO score groups, but for some popular genes in bladder cancer studies such as TP53, RB1, and FGFR3, the mutation frequency difference between high and low SUMO score groups was more than 10%. TP53 and RB1 were important tumor suppressive genes and studies have found they were regulated by PTMs such as SUMOylation, which could lead to abnormal proliferation and tumorigenesis (44). FGFR3 gene aberrations were common in bladder cancer and FGFR3 signaling was reported to promote epithelial-to-mesenchymal transition (EMT) (45). But no previous studies have reported the relationship between SUMOylation and FGFR3, so further investigations were needed in this field. Nevertheless, we found the combination of TMB and SUMO score could excellently predict the clinical outcome of the patients with BCa (**Figure 4H**). Patients with high TMB and low SUMO score had the greatest survival advantage while those with low TMB and high SUMO score had the worst. All these results revealed SUMO score was a considerable predictive biomarker independent of TMB for the prognosis of patients with BCa.

**Table 2:** Mutant genes between high and low SUMO score groups.

gene	H-wild	H-mutation	L-wild	L-mutation	p-value
FGFR3	188 (94%)	12 (6%)	158 (77.83%)	45 (22.17%)	6.37E−06
RB1	156 (78%)	44 (22%)	188 (92.61%)	15 (7.39%)	6.14E−05
TP53	88 (44%)	112 (56%)	129 (63.55%)	74 (36.45%)	0.000125249
KDM6A	169 (84.5%)	31 (15.5%)	146 (71.92%)	57 (28.08%)	0.003330535
NFE2L2	182 (91%)	18 (9%)	198 (97.54%)	5 (2.46%)	0.008958805
MYO9A	195 (97.5%)	5 (2.5%)	186 (91.63%)	17 (8.37%)	0.017495641
ERBB2	171 (85.5%)	29 (14.5%)	189 (93.1%)	14 (6.9%)	0.02085486
DNAH8	190 (95%)	10 (5%)	179 (88.18%)	24 (11.82%)	0.02233436
ELF3	185 (92.5%)	15 (7.5%)	173 (85.22%)	30 (14.78%)	0.030667654
UTRN	191 (95.5%)	9 (4.5%)	182 (89.66%)	21 (10.34%)	0.040835835
UTP20	184 (92%)	16 (8%)	197 (97.04%)	6 (2.96%)	0.044495421

We also observed that a remarkable relationship existed between SUMO score and immune cell infiltration in the TME (**Figure 4I**). SUMO score was positively correlated to the enrichment of immune cells such as activated B cell, activated CD4^+^ T cell, activated CD8^+^ T cell, DC, MDSC, NKT cell, Th1, Th2, and Treg, which was in accordance with the results in SUMOylation patterns (**Figure 2H**) and indicated SUMO score could finely evaluate TME cell-infiltration characterization in individuals.

### Characteristics of SUMOylation in Immunotherapy and Chemotherapy

Overall, patients with low SUMO score had better response to immunotherapies (**Supplementary Figures 3C, D**). We also found patients in the high SUMO group had higher Tumor Immune Dysfunction and Exclusion (TIDE) score (**Figure 5C**), which could predict the response to ICB immunotherapy (24). Then we further explored whether the SUMO score had a predictive significance for the outcome of immunotherapies with different kinds of ICBs. As shown in **Figure 5D**, we evaluated the interaction between SUMO score and the response to anti-PD-1 and anti-CTLA-4 immunotherapy. We found low SUMO score was related to better response to anti-PD-1 immunotherapy. After Bonferroni correction, there still existed a remarkable correlation between low SUMO score and response to anti-PD-1 immunotherapy although lacked statistical significance. Then we divided the patients into four subgroups according to the use of anti-CTLA-4 and anti-PD-1 immunotherapies: CTLA-4 negative PD-1 negative (**Figure 5E**), CTLA-4 negative PD-1 positive (**Figure 5F**), CTLA-4 positive PD-1 negative (**Figure 5G**) and CTLA-4 positive PD-1 positive (**Figure 5H**). In CTLA-4 positive PD-1 negative and CTLA-4 negative PD-1 negative subgroups, low SUMO score was related to better immunotherapy response and prognosis, while in CTLA-4 positive PD-1 positive and CTLA-4 negative PD-1 positive subgroups the results were exactly opposite, which further proved that SUMOylation had a tighter relationship with immunotherapy targeted at PD-1 compared to other ICBs and patients with low SUMO score would benefit more from anti-PD-1 immunotherapy.

Anti-PD-L1 immunotherapy has been proven effective for patients with metastatic urothelial carcinoma in a multicenter, single-arm phase 2 trial using atezolizumab (IMvigor 210, NCT02108652) (20). Using the data acquired from IMvigor 210 cohort, we further verified the interaction between SUMO score and response to immunotherapies. Patients could be divided into three different immune phenotypes according to their response to immunotherapies: the immune-inflamed phenotype, the immune-excluded phenotype and the immune-desert phenotype (46). The immune-inflamed phenotype was characterized by enrichment of various immune cells like CD4^+^ T cells, CD8^+^ T cells, and monocytic cells in the tumor parenchyma, as well as many proinflammatory and effector cytokines. Clinical responses to immunotherapies usually occurred in patients with this immune phenotype. The immune-excluded phenotype was also characterized by abundance in various kinds of immune cells, however, not in the tumor parenchyma, but in the stroma. So clinical responses to immunotherapies were uncommon in patients with this phenotype. The immune-desert phenotype was characterized by lack of T cells in both the tumor parenchyma and stroma, so it is not surprising that patients with this phenotype rarely responded to immunotherapies (46). Therefore, we further investigated the relationship between SUMO score and immune phenotypes. As shown in **Figure 5I**, the proportion of the immune-excluded phenotype was considerable between the high and low SUMO score groups, but the high SUMO score group was significantly enriched in the immune-desert phenotype and the low SUMO score group was dramatically enriched in the immune-inflamed phenotype. Similarly, we also found patients with the immune-desert phenotype had the highest SUMO score and patients with the immune-desert phenotype had the lowest (**Figure 5J**). As immune phenotype was important to predict the response to immunotherapies, we next explored whether SUMO score was related to response to anti-PD-L1 immunotherapy. Not surprisingly, patients in the low SUMO score group showed significant therapeutic advantages and clinical response to immunotherapy (**Figures 5K, L**). When combining complete response and partial response, as well as stable disease and progressive disease to create a binary outcome, the differences of SUMO score among patients with different main responses were more evidenced (**Figures 5M, N**). All these results revealed SUMO score could serve as a latent indicator for predicting the response to immunotherapies.

**Figure 6:**
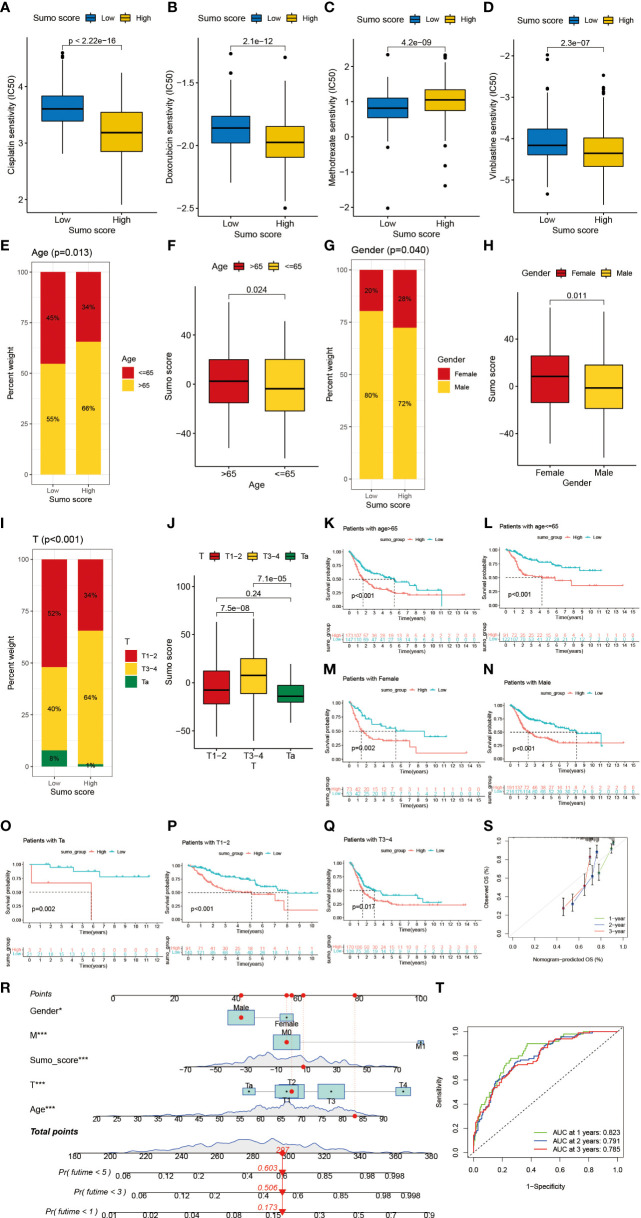
The role of SUMOylation patterns in chemotherapy and tumor clinicopathological characteristics and the construction of a nomogram using SUMO score to predict prognosis in clinical scenarios. **(A–D)** Differences in IC50 of chemotherapy drugs between high and low SUMO score groups in meta-cohort. **(A)** cisplatin (P <0.001, Wilcoxon test). **(B)** doxorubicin (P <0.001, Wilcoxon test). **(C)** methotrexate (P <0.001, Wilcoxon test). **(D)** vinblastine (P <0.001, Wilcoxon test). **(E)** The proportion of patients with different age in low or high SUMO score groups. Age ≤65/age >65: 45%/55% in the low SUMO score groups and 34%/66% in the high SUMO score groups. **(F)** Differences in SUMO score between age >65 and age ≤65 subgroups in meta-cohort (P = 0.024, Wilcoxon test). **(G)** The proportion of patients with different gender in low or high SUMO score groups. Female/male: 20%/80% in the low SUMO score groups and 28%/72% in the high SUMO score groups. **(H)** Differences in SUMO score between female and male subgroups in meta-cohort (P = 0.011, Wilcoxon test). **(F)** Differences in SUMO score between age >65 and age ≤65 subgroups in meta-cohort (P = 0.024, Wilcoxon test). **(I)** The proportion of patients with different T category in low or high SUMO score groups. T1–2/T3–4/Ta: 52%/40%/8% in the low SUMO score groups and 34%/64%/1% in the high SUMO score groups. **(J)** Differences in SUMO score among T1–2, T3–4 and Ta subgroups in meta-cohort (Kruskal–Wallis H test). **(K–S)** Survival analyses for high and low SUMO score groups in subgroups of meta-cohort using Kaplan–Meier curves. **(K)** Patients >65 subgroup (P <0.001, Log-rank test). **(L)** Patients ≤65 subgroup (P <0.001, Log-rank test). **(M)** Female subgroup (P = 0.002, Log-rank test). **(N)** Male subgroup (P <0.001, Log-rank test). **(O)** Patients with Ta stage tumor subgroup (P = 0.002, Log-rank test). **(P)** Patients with T1–2 stage tumor subgroup (P <0.001, Log-rank test). **(Q)** Patients with T3–4 stage tumor subgroup (P = 0.017, Log-rank test). **(R)** Nomogram for patients predicting survival outcome for 1, 3, and 5 years. Gender, M category, SUMO score, T category and age were marked as “points”. Total points by adding the five points could predict survival probability. One patient who was male and had tumor with T2M0 category was randomly selected for analysis. After adding the score of each item, a total score of 297 and the corresponding survival probability for 1, 3, and 5 years respectively were obtained. **(S)** Calibration curves of nomograms in terms of the agreement between predicted and observed 1, 3, and 5 years of outcomes in meta-cohort. **(T)** Receiver operating characteristic (ROC) curve. The area under curve (AUC) for 1, 3, and 5 years was 0.823, 0.791, and 0.785 respectively, which showed a favorable ability of discrimination.

Next, we would like to investigate the relationship between SUMO score and response to chemotherapy. We screened out several commonly used drugs for intravesical chemotherapy in NMIBC such as doxorubicin, mitomycin C, and gemcitabine (10) and for adjuvant treatment after surgery in MIBC such as cisplatin, methotrexate, and vinblastine (11). We found that high SUMO score was related with low half maximal inhibitory concentration (IC50) in cisplatin, doxorubicin, mitomycin C, vinblastine and gemcitabine (**Figures 6A, B, D** and **Supplementary Figures 3E, F**), which means higher sensitivity to chemotherapy, but methotrexate was just the reverse (**Figure 6C**). We also found significant relationship between SUMO score and other drugs (**Supplementary Figures 5G–N**), some of which were newly developed and have not been put into clinical use to treat bladder cancer, thus we could screen out appropriate drugs according to the SUMOylation patterns of patients. In summary, the above results showed a unique role of SUMO score to predict the efficacy of immunotherapy and chemotherapy and could be used to guide clinical treatment.

### Characteristics of Clinical Traits in SUMOylation-Related Phenotypes and the Construction of a Nomogram Using SUMO Score to Predict Prognosis in Clinical Scenarios

Then we lucubrated the interaction between SUMO score and clinical signatures and found SUMO score was significantly related to age (**Figures 6E, F**), gender (**Figures 6G, H**), grade (**Supplementary Figures 5G, H**), T category (**Figures 6I, J**), M category (**Supplementary Figures 5C, D**) and final survival status (**Supplementary Figures 5A, B**). However, the distribution difference of SUMO score in N category did not show a statistical significance (**Supplementary Figures 5E, F**). In addition, we performed subgroup analyses and found SUMO score was a good predictor of survival in all subgroups divided by age (>65 and ≤65) (**Figures 6K, L**), gender (female and male) (**Figures 6M, N**), grade (high and low grade) (**Supplementary Figures 5O, P**) and T category (Ta, T1–2, T3–4) (**Figures 6O–Q**). But for subgroups divided by M and N categories, SUMO score was only significantly related to patients with M0 and N0 category (**Supplementary Figures 5I, L**).

Given the dramatic significance of SUMO score in predicting the prognosis of patients with BCa, we would like to establish a clinical predictive model to predict the survival probability of patients with BCa at 1, 3, and 5 years based on the clinical characteristics of the patients in the meta-cohort. First, we screened out five clinical features, namely, SUMO score, age, gender, T category and M category using univariate and multivariate COX regression model (**Supplementary Figures 5Q, R**). Then we constructed a nomograph based on these five clinical features which were easily accessible (**Figure 6R**). The C-indexes for 1, 3, and 5 years were 0.823, 0.791, and 0.785 respectively, which indicated that the nomogram had a good predictive value (**Figures 6S, T**).

## Discussion

Nowadays, more and more studies have focused on the interaction between SUMOylation and tumorigenesis, metastasis, and progression (13), including bladder cancer (16–19). As described above, many SUMOylation related proteins were overexpressed in tumor tissues, so drugs targeted at these aberrantly expressed components in SUMOylation process would be a latent treatment for cancer in the future. For example, ginkgolic acid, which could inhibit SAE1 subunit of SUMO E1-activating enzyme, was reported to suppress the growth of NOTCH1-activated breast epithelial cells and induce apoptosis (47). Treatment targeted at SUMOylation could not only directly impair the growth and survival of tumor, but also contribute to overcome the tumor heterogeneity and provide benefit to immunotherapy (16).

In the last decade, immunotherapies with ICBs targeted at PD-1 and PD-L1 were developed rapidly in BCa. Clinical trials using atezolizumab (20), nivolumab (48) and pembrolizumab (49) have all reported promising results, and immunotherapy has gradually become a new choice for those with high grade and metastatic disease (11). However, only approximately 20% of the patients could respond to ICB treatment because of the molecular heterogeneity of BCa, so it is important for us to find out biomarkers of genomic signatures to predict and select the patients who might have favorable outcomes during immunotherapies. Similarly, some biomarkers could also help in stratifying patients to predict the response to chemotherapy. For example, excision repair cross complementing 1 (ERCC1) has been considered as a prognostic biomarker to predict the sensitivity to cisplatin-based chemotherapy in patients with BCa (50). Therefore, it must be intriguing and urgent to establish a panel of validated biomarkers for response to chemotherapy and immunotherapy in the future.

Although previous studies have inquired the critical roles SUMOylation played in BCa, however, most of them paid more attention to limited SUMOylation related proteins and did not go deep into the comprehensive effect of SUMOylation in BCa and the interaction between TME cell infiltration and SUMOylation, which was necessary to guide more effective immunotherapy, chemotherapy and therapies targeted at SUMOylation.

Here in this article, we first investigated the differences in activated pathways related to SUMOylation between tumor and normal adjacent tumor tissue. Consistent with previous studies, we found all these pathways were more activated in tumor than in normal adjacent tumor tissue and the differences were significant. Then we combined the GEO dataset GSE13507 and TGCA-BLCA cohort into a new meta-cohort, and divided the patients with BCa in the meta-cohort into two SUMOylation patterns termed SUMO cluster C1 and C2 using NMF clustering according to the expression level of these SUMOylation related genes. Surprisingly, although we did not find significant differences in clinical characteristics, remarkable differences were discovered in the overall survival of patients and TME immune cell infiltration between the two SUMO clusters. It was interesting that SUMO cluster C2 was enriched in almost all kinds of immune cells but did not show a corresponding survival advantage, where we found MDSC and Treg might play pivotal roles. MDSC and Treg were important immunosuppressive cells which could contribute to immune evasion, tumor angiogenesis, drug resistance, and tumor metastases (51, 52) and lead to poor prognosis. Many previous studies have demonstrated SUMOylation was important to the stability and normal function of MDSC and Treg. Yu et al. reported that SENP3 could mediate the deSUMOylation of BACH2 and inhibit its nuclear export, thus being critical to regulate functions of Treg. Treg cell-specific deletion of Senp3 could result in excessive inflammatory response (53). Studies also found SENP1 regulated the development and function of MDSC and the loss of SENP1 could increase SUMOylation of CD45, promote MDSC development and lead to tumorigenesis (54). Therefore, it is conceivable that the TME immune cell infiltration is tightly related to SUMOylation patterns. We also performed a GSVA enrichment analysis to dig out hallmark pathways and biological processes behind SUMOylation patterns, and we found SUMO cluster C2 showed enrichment in pathways related to cell proliferation and tumorigenesis, which was in accordance with the poor prognosis.

Next, we used univariate COX regression to screen out 1,934 SUMOylation related DEGs with prognostic value and classified the patients into two distinct genomic subtypes named gene cluster A and B using unsupervised clustering based on the expression level of these genes. We found patients in gene cluster A had better survival outcome and the gene clusters were tightly connected with SUMOylation patterns. We also performed a GSVA enrichment analysis between the two distinct gene clusters gene and found cluster A was also remarkably enriched in pathways associated with metabolism, while gene cluster B exhibited enrichment in pathways linked to cell cycle and immune response to infection, which was approximately in accordance with the results in SUMOylation patterns. Interestingly, we found PPAR signaling was significantly activated in gene cluster A, but the previous studies have reported PPARγ signaling could increase the incidence and progression of bladder cancer, which might lead to worse prognosis (37, 38). So, it remains unclear what specific role PPARγ signaling plays in the development and prognosis of bladder cancer.

Furthermore, given the individual heterogeneity and complexity of SUMOylation, it was necessary for us to explore the latent SUMOylation pattern in individuals. Therefore, we constructed a set of scoring system named SUMO score to quantify the SUMOylation pattern of individual patients with bladder cancer. We found significant differences in SUMO score between different gene clusters and SUMO clusters. SUMO cluster C2 and gene cluster B showed higher SUMO score. Similarly, patients with low SUMO score exhibited a significant survival advantage. As TMB has been demonstrated as an effective biomarker to predict the response to treatment with ICBs in BCa (43), we further explored the relationship between TMB and SUMO score. However, we did not find significant correlation between TMB and SUMO score and there were also no obvious distribution differences of TMB in most mutated genes between low and high SUMO score groups. But we found remarkable survival differences when combining SUMO score and TMB to divide the patients into four distinct subgroups, which indicated that SUMO score might be a potent predictive biomarker independent of TMB for the prognosis of patients with BCa. We also observed there existed a remarkable relationship between SUMO score and immune cell infiltration in the TME, which was consistent with the results in SUMOylation patterns and implied SUMO score could finely evaluate TME cell-infiltration characterization in individuals.

Many patients have benefited from immunotherapy using ICBs such as PD-1, PD-L1 and CTLA-4 blockade, but many more patients did not see pronounced clinical response to immunotherapeutic intervention (55). In patients with BCa, PD-1/PD-L1 blockade has demonstrated significant benefit in patients with unresectable and metastatic BC in the second-line setting, either as monotherapy or in combination with chemotherapy or CTLA-4 checkpoint inhibition (11). The results of the phase II trial using the PD-1 inhibitor pembrolizumab reported a complete pathological remission (pT0) in 42% and pathological response (<pT2) in 54% of patients (49), whereas another single-arm phase II trial with atezolizumab showed a pathologic complete response rate of 31% (20). These results suggested that the response rate was still low and it was important to screen out patients who were appropriate for immunotherapy. Our results found lower SUMO score was connected with better response to immunotherapy using PD-1 blockade. Next, using the data acquired from IMvigor 210 (NCT02108652) cohort, we further verified the interaction between SUMO score and response to immunotherapy. Not surprisingly, we also found low SUMO score was connected better response to anti-PD-L1 treatment. Besides, we discovered the immune phenotype was also tightly linked to SUMO score and low SUMO score group was dramatically enriched in the immune-inflamed phenotype, which indicated a better response to immunotherapy. Therefore, we showed that SUMOylation patterns played a nonnegligible role in distinguishing different TME and could be an effective biomarker to select patients who were appropriate for immunotherapy.

Moreover, we also explored the relationship between SUMO score and response to chemotherapy. Mitomycin C (MMC), interferon (IFN), adriamycin, epirubicin, and gemcitabine (GEM) were commonly used drugs for intravesical chemotherapy after TURB apart from Bacille Calmette–Guérin (BCG) to prevent recurrence in patients with NMIBC (56). The traditional chemotherapy for MIBC followed a regimen of methotrexate, vinblastine, doxorubicin, and cisplatin (MVAC), and was gradually replaced by a new plan including gemcitabine plus cisplatin (GC) (12). Therefore, we evaluated the relationship between SUMO score and sensitivity to commonly used chemotherapy drugs and found IC50 for all these drugs exhibited a significant difference between high and low SUMO score group, which indicated a unique role of SUMO score to predict the efficacy of chemotherapy and guide clinical treatment.

Finally, we lucubrated the interaction between SUMO score and clinical signatures and found SUMO score was significantly related to age, gender, grade, T category, M category, and final survival status. We also performed subgroup survival analyses and found SUMO score was a good predictor of survival. Then we constructed a nomograph based on five features, namely, SUMO score, age, gender, T category, and M category to predict the survival probability of patients with BCa in 1, 3, and 5 years, respectively. The calibration curve and AUC of ROC curve showed a favorable ability of discrimination.

In general, our study provided a comprehensive insight into the interaction between SUMOylation, tumor clinicopathological characteristics, TMB, TME immune cell infiltration, and response to chemotherapy and immunotherapy. We demonstrated that different SUMOylation patterns could contribute to distinguishing the landscape of TME immune cell infiltration and clinical characteristics among patients, which was further verified using SUMO score within individuals. Moreover, SUMO score could also function as a predictive indicator for the survival of patients independent of TMB. Finally, we also evaluated the ability of SUMO score to predict the response to immunotherapy using ICBs and chemotherapy, which might help in improving therapeutic strategies, screening patients eligible for immunotherapy or chemotherapy and guiding individual precision therapy in the future.

However, we also realize that there still exist several shortcomings and limitations in our study. First, as many new SUMOylation related proteins were discovered recently, the SUMOylation related genes which we collected and used for analyses might not be comprehensive enough, which would bring some bias into our studies. Second, the current omics data only provide the level of mRNA but the SUMOylation process relies on proteins, which will bring in some inaccuracies. Third, the number of clinical samples is limited and our study is lack of verification from other clinical data sets apart from the public data which will be helpful to further confirm our conclusions, and whether SUMOylation has a similar role in other types of cancer has not been verified. Therefore, we are prepared to collect some clinical samples to further verify our conclusions, and assess the role of SUMOylation in other urinary system tumors. Finally, the specific mechanisms in the interaction between SUMOylation patterns and TMB immune cell infiltration remain unclear and need further studies.

In conclusion, our work demonstrated and interpreted the complicated regulation mechanisms of SUMOylation in bladder cancer. The differences in SUMOylation patterns in population or individuals could significantly influence the heterogeneity in tumor clinicopathological features and TME, thus influencing the response to immunotherapy and chemotherapy. Therefore, better understanding and evaluating SUMOylation patterns could be beneficial in selecting appropriate patients, guiding clinical therapeutic strategy and improving the prognosis of patients with BCa.

## Data Availability Statement

The datasets presented in this study can be found in online repositories. The names of the repository/repositories and accession number(s) can be found in the article/**Supplementary Material**.

## Author Contributions

Q-DX and J-XS analyzed the data, wrote the manuscript and drew the figures. S-GW, ZL, and JH designed the study. YX, JX, C-QL, J-ZX, YA, and M-YX contributed to the critical revision of the manuscript. Q-DX, J-XS, JH, and S-GW contributed equally to this work. All authors listed have made a substantial, direct, and intellectual contribution to the work and approved it for publication.

## Funding

This work was supported by the Natural Science Foundation of China (81772729).

## Conflict of Interest

The authors declare that the research was conducted in the absence of any commercial or financial relationships that could be construed as a potential conflict of interest.

## Publisher’s Note

All claims expressed in this article are solely those of the authors and do not necessarily represent those of their affiliated organizations, or those of the publisher, the editors and the reviewers. Any product that may be evaluated in this article, or claim that may be made by its manufacturer, is not guaranteed or endorsed by the publisher.

## Supplementary Material

Supplementary Figure 1 The process of generating two distinct SUMOylation patterns using NMF. **(A)** When ranks from 2 to 10, there were changes in several cluster results related parameters such as the cophenetic correlation, residual sum of squares (RSS), and silhouette distribution et al. **(B)** Connectivity matrix for patients with bladder cancer in the meta-cohort by NMF when k ranged from 2 to 10.

Supplementary Figure 2 The process of generating two distinct SUMO cluster related DEGs genomic patterns. **(A–H)** Unsupervised clustering of 1934 SUMO cluster related DEGs in meta-cohort and consensus matrices for k = 1, 3 - 9. **(I)** The cumulative distribution function (CDF) curve for k = 2 - 9. **(J)** The scree plot for k = 2 - 9. K The track plot for k = 2 - 9.

Supplementary Figure 3 The role of SUMOylation patterns in survival outcome and response to immunotherapy and chemotherapy. **(A)** Survival analyses for low (202 cases) and high (201 cases) SUMO score patient groups in TCGA-BLCA cohort using Kaplan-Meier curves (P < 0.001, Log-rank test). **(B)** Survival analyses for low (92 cases) and high (73 cases) SUMO score patient groups in GSE13507 cohort using Kaplan-Meier curves (P < 0.001, Log-rank test). **(C)** Differences in SUMO score between immunotherapy response and nonresponse groups in TCGA-BLCA cohort (P < 0.001, Wilcoxon test). **(D)** The proportion of patients who response to immunotherapy in low or high SUMO score groups. **(E–N)** Differences in IC50 of chemotherapy drugs between high and low SUMO score groups in meta-cohort. € Gemcitabine (P = 0.002, Wilcoxon test). **(F)** Mitomycin C (P = 0.03, Wilcoxon test). **(G)** Bicalutamide (P < 0.001, Wilcoxon test). **(H)** Bleomycin (P < 0.001, Wilcoxon test). **(I)** Bosutinib (P < 0.001, Wilcoxon test). **(J)** Dasatinib (P < 0.001, Wilcoxon test). **(K)** Epothilone (P < 0.001, Wilcoxon test). **(L)** Parthenolide (P < 0.001, Wilcoxon test). **(M)** Tipifarnib (P < 0.001, Wilcoxon test). **(N)** Vinorelbine (P < 0.001, Wilcoxon test).

Supplementary Figure 4 Survival analyses for patients with high and low SUMO score in external validation dataset GSE69795 and GSE70691. **(A)** Survival analyses for low (13 cases) and high (25 cases) SUMO score patient groups in GSE69795 cohort using Kaplan-Meier curves (P = 0.183, Log-rank test). **(B)** Survival analyses for low (12 cases) and high (37 cases) SUMO score patient groups in GSE70691 cohort using Kaplan-Meier curves (P = 0.074, Log-rank test).

Supplementary Figure 5 Survival analyses for patients with high and low SUMO score in several subgroups. **(A)** The proportion of patients with different final survival status in low or high SUMO score groups. Alive/dead: 68%/32% in the low SUMO score groups and 45%/55% in the high SUMO score groups. **(B)** Differences in SUMO score between alive and dead subgroups in meta-cohort (P < 0.001, Wilcoxon test). **(C)** Differences in SUMO score among M0, M1 and MX groups in meta-cohort (Kruskal-Wallis H test). **(D)** The proportion of patients with M0, M1 and MX stage tumor in low or high SUMO score groups. € Differences in SUMO score among N0, N1-N3 and NX groups in meta-cohort (Kruskal-Wallis H test). **(F)** The proportion of patients with N0, N1-N3 and NX stage tumor in low or high SUMO score groups. **(G)** The proportion of patients with different tumor grade in low or high SUMO score groups. High grade/low grade: 62%/38% in the low SUMO score groups and 91%/9% in the high SUMO score groups. **(H)** Differences in SUMO score between high and low tumor grade groups in meta-cohort (P < 0.001, Wilcoxon test). **(I-P)** Survival analyses for high and low SUMO score groups in subgroups of meta-cohort using Kaplan-Meier curves. **(I)** Patients with M0 stage tumor subgroup (P < 0.001, Log-rank test). **(J)** Patients with M1 stage tumor subgroup (P = 0.646, Log-rank test). **(K)** Patients with MX stage tumor subgroup (P = 0.090, Log-rank test). **(L)** Patients with N0 stage tumor subgroup (P < 0.001, Log-rank test). **(M)** Patients with N1-3 stage tumor subgroup (P = 0.083, Log-rank test). **(N)** Patients with NX stage tumor subgroup (P = 0.031, Log-rank test). **(O)** Patients with high grade tumor subgroup (P < 0.001, Log-rank test). **(P)** Patients with low grade tumor subgroup (P < 0.001, Log-rank test). **(Q)** Univariate Cox regression analyses of OS in meta-cohort. The p-values were obtained by univariate Cox regression. **(R)** Multivariate Cox regression analyses of OS in meta-cohort. The p-values were obtained by multivariate Cox regression.

## References

[1] Venne AS Kollipara L Zahedi RP. The Next Level of Complexity: Crosstalk of Posttranslational Modifications. Proteomics (2014) 14(4-5):513–24. doi: 10.1002/pmic.201300344 24339426

[2] Han ZJ Feng YH Gu BH Li YM Chen H. The Post-Translational Modification, Sumoylation, and Cancer (Review). Int J Oncol (2018) 52(4):1081–94. doi: 10.3892/ijo.2018.4280 PMC5843405 29484374

[3] Matunis MJ Coutavas E Blobel G. A Novel Ubiquitin-Like Modification Modulates the Partitioning of the Ran-Gtpase-Activating Protein Rangap1 Between the Cytosol and the Nuclear Pore Complex. J Cell Biol (1996) 135(6 Pt 1):1457–70. doi: 10.1083/jcb.135.6.1457 PMC2133973 8978815

[4] Hickey CM Wilson NR Hochstrasser M. Function and Regulation of SUMO Proteases. Nat Rev Mol Cell Biol (2012) 13(12):755–66. doi: 10.1038/nrm3478 PMC3668692 23175280

[5] Yang Y He Y Wang X Liang Z He G Zhang P. Protein Sumoylation Modification and its Associations With Disease. Open Biol (2017) 7(10):170167. doi: 10.1098/rsob.170167 29021212 PMC5666083

[6] Flotho A Melchior F. Sumoylation: A Regulatory Protein Modification in Health and Disease. Annu Rev Biochem (2013) 82:357–85. doi: 10.1146/annurev-biochem-061909-093311 23746258

[7] Richters A Aben KKH Kiemeney L. The Global Burden of Urinary Bladder Cancer: An Update. World J Urol (2020) 38(8):1895–904. doi: 10.1007/s00345-019-02984-4 PMC7363726 31676912

[8] Antoni S Ferlay J Soerjomataram I Znaor A Jemal A Bray F. Bladder Cancer Incidence and Mortality: A Global Overview and Recent Trends. Eur Urol (2017) 71(1):96–108. doi: 10.1016/j.eururo.2016.06.010 27370177

[9] Sloan FA Yashkin AP Akushevich I Inman BA. The Cost to Medicare of Bladder Cancer Care. Eur Urol Oncol (2020) 3(4):515–22. doi: 10.1016/j.euo.2019.01.015 31412015

[10] Babjuk M Burger M Compérat EM Gontero P Mostafid AH Palou J. European Association of Urology Guidelines on Non-Muscle-Invasive Bladder Cancer (Tat1 and Carcinoma in Situ) - 2019 Update. Eur Urol (2019) 76(5):639–57. doi: 10.1016/j.eururo.2019.08.016 31443960

[11] Witjes JA Bruins HM Cathomas R Compérat EM Cowan NC Gakis G. European Association of Urology Guidelines on Muscle-Invasive and Metastatic Bladder Cancer: Summary of the 2020 Guidelines. Eur Urol (2021) 79(1):82–104. doi: 10.1016/j.eururo.2020.03.055 32360052

[12] Lobo N Mount C Omar K Nair R Thurairaja R Khan MS. Landmarks in the Treatment of Muscle-Invasive Bladder Cancer. Nat Rev Urol (2017) 14(9):565–74. doi: 10.1038/nrurol.2017.82 28675174

[13] Eifler K Vertegaal ACO. Sumoylation-Mediated Regulation of Cell Cycle Progression and Cancer. Trends Biochem Sci (2015) 40(12):779–93. doi: 10.1016/j.tibs.2015.09.006 PMC4874464 26601932

[14] Mo YY Yu Y Theodosiou E Ee PLR Beck WT. A Role for Ubc9 in Tumorigenesis. Oncogene (2005) 24(16):2677–83. doi: 10.1038/sj.onc.1208210 15735760

[15] Moschos SJ Jukic DM Athanassiou C Bhargawa R Dacic S Wang X. Expression Analysis of Ubc9, the Single Small Ubiquitin-Like Modifier (SUMO) E2 Conjugating Enzyme, in Normal and Malignant Tissues. Hum Pathol (2010) 41(9):1286–98. doi: 10.1016/j.humpath.2010.02.007 20561671

[16] Oo HZ Seiler R Black PC Daugaard M. Post-Translational Modifications in Bladder Cancer: Expanding the Tumor Target Repertoire. Urol Oncol (2020) 38(12):858–66. doi: 10.1016/j.urolonc.2018.09.001 30342880

[17] Tan M Gong H Wang J Tao L Xu D Bao E. SENP2 Regulates MMP13 Expression in a Bladder Cancer Cell Line Through Sumoylation of TBL1/TBLR1. Sci Rep (2015) 5:13996. doi: 10.1038/srep13996 26369384 PMC4570209

[18] Tan M Zhang D Zhang E Xu D Liu Z Qiu J. SENP2 Suppresses Epithelial-Mesenchymal Transition of Bladder Cancer Cells Through Desumoylation of TGF-βri. Mol Carcinog (2017) 56(10):2332–41. doi: 10.1002/mc.22687 28574613

[19] Chen C Zheng H Luo Y Kong Y An M Li Y. Sumoylation Promotes Extracellular Vesicle-Mediated Transmission of Lncrna ELNAT1 and Lymph Node Metastasis in Bladder Cancer. J Clin Invest (2021) 131(8):e146431. doi: 10.1172/JCI146431 PMC8262506 33661764

[20] Rosenberg JE Hoffman-Censits J Powles T van der Heijden MS Balar AV Necchi A. Atezolizumab in Patients With Locally Advanced and Metastatic Urothelial Carcinoma Who Have Progressed Following Treatment With Platinum-Based Chemotherapy: A Single-Arm, Multicentre, Phase 2 Trial. Lancet (2016) 387(10031):1909–20. doi: 10.1016/S0140-6736(16)00561-4 PMC5480242 26952546

[21] Mariathasan S Turley S Nickles D Castiglioni A Yuen K Wang Y. Tgfβ Attenuates Tumour Response to PD-L1 Blockade by Contributing to Exclusion of T Cells. Nature (2018) 554(7693):544–8. doi: 10.1038/nature25501 PMC6028240 29443960

[22] Charoentong P Finotello F Angelova M Mayer C Efremova M Rieder D. Pan-Cancer Immunogenomic Analyses Reveal Genotype-Immunophenotype Relationships and Predictors of Response to Checkpoint Blockade. Cell Rep (2017) 18(1):248–62. doi: 10.1016/j.celrep.2016.12.019 28052254

[23] Subramanian A Tamayo P Mootha VK Mukherjee S Ebert BL Gillette MA. Gene Set Enrichment Analysis: A Knowledge-Based Approach for Interpreting Genome-Wide Expression Profiles. Proc Natl Acad Sci USA (2005) 102(43):15545–50. doi: 10.1073/pnas.0506580102 PMC1239896 16199517

[24] Jiang P Gu S Pan D Fu J Sahu A Hu X. Signatures of T Cell Dysfunction and Exclusion Predict Cancer Immunotherapy Response. Nat Med (2018) 24(10):1550–8. doi: 10.1038/s41591-018-0136-1 PMC6487502 30127393

[25] Hoshida Y Brunet JP Tamayo P Golub TR Mesirov JP. Subclass Mapping: Identifying Common Subtypes in Independent Disease Data Sets. PLoS One (2007) 2(11):e1195. doi: 10.1371/journal.pone.0001195 18030330 PMC2065909

[26] Geeleher P Cox N Huang RS. Prrophetic: An R Package for Prediction of Clinical Chemotherapeutic Response From Tumor Gene Expression Levels. PLoS One (2014) 9(9):e107468. doi: 10.1371/journal.pone.0107468 25229481 PMC4167990

[27] Czuba LC Hillgren KM Swaan PW. Post-Translational Modifications of Transporters. Pharmacol Ther (2018) 192:88–99. doi: 10.1016/j.pharmthera.2018.06.013 29966598 PMC6263853

[28] Patwardhan A Cheng N Trejo J. Post-Translational Modifications of G Protein-Coupled Receptors Control Cellular Signaling Dynamics in Space and Time. Pharmacol Rev (2021) 73(1):120–51. doi: 10.1124/pharmrev.120.000082 PMC7736832 33268549

[29] Hill SM Wrobel L Rubinsztein DC. Post-Translational Modifications of Beclin 1 Provide Multiple Strategies for Autophagy Regulation. Cell Death Differ (2019) 26(4):617–29. doi: 10.1038/s41418-018-0254-9 PMC6460389 30546075

[30] Liu J Qian C Cao X. Post-Translational Modification Control of Innate Immunity. Immunity (2016) 45(1):15–30. doi: 10.1016/j.immuni.2016.06.020 27438764

[31] Zhao X. SUMO-Mediated Regulation of Nuclear Functions and Signaling Processes. Mol Cell (2018) 71(3):409–18. doi: 10.1016/j.molcel.2018.07.027 PMC6095470 30075142

[32] Kumar V Patel S Tcyganov E Gabrilovich DI. The Nature of Myeloid-Derived Suppressor Cells in the Tumor Microenvironment. Trends Immunol (2016) 37(3):208–20. doi: 10.1016/j.it.2016.01.004 PMC4775398 26858199

[33] Hinshaw DC Shevde LA. The Tumor Microenvironment Innately Modulates Cancer Progression. Cancer Res (2019) 79(18):4557–66. doi: 10.1158/0008-5472.CAN-18-3962 PMC6744958 31350295

[34] von Boehmer H Daniel C. Therapeutic Opportunities for Manipulating T(Reg) Cells in Autoimmunity and Cancer. Nat Rev Drug Discov (2013) 12(1):51–63. doi: 10.1038/nrd3683 23274471

[35] Quail DF Joyce JA. Microenvironmental Regulation of Tumor Progression and Metastasis. Nat Med (2013) 19(11):1423–37. doi: 10.1038/nm.3394 PMC3954707 24202395

[36] Belfiore A Genua M Malaguarnera R. Ppar-γ Agonists and Their Effects on IGF-I Receptor Signaling: Implications for Cancer. PPAR Res (2009) 2009:830501. doi: 10.1155/2009/830501 19609453 PMC2709717

[37] Chiu M McBeth L Sindhwani P Hinds TD. Deciphering the Roles of Thiazolidinediones and Pparγ in Bladder Cancer. PPAR Res (2017) 2017:4810672. doi: 10.1155/2017/4810672 28348577 PMC5350343

[38] Lewis JD Habel LA Quesenberry CP Strom BL Peng T Hedderson MM. Pioglitazone Use and Risk of Bladder Cancer and Other Common Cancers in Persons With Diabetes. Jama (2015) 314(3):265–77. doi: 10.1001/jama.2015.7996 26197187

[39] Hsiao FY Hsieh PH Huang WF Tsai YW Gau CS. Risk of Bladder Cancer in Diabetic Patients Treated With Rosiglitazone or Pioglitazone: A Nested Case–Control Study. Drug Saf (2013) 36(8):643–9. doi: 10.1007/s40264-013-0080-4 23797604

[40] Yang DR Lin SJ Ding XF Miyamoto H Messing E Li LQ. Higher Expression of Peroxisome Proliferator-Activated Receptor γ or its Activation by Agonist Thiazolidinedione-Rosiglitazone Promotes Bladder Cancer Cell Migration and Invasion. Urology (2013) 81(5):1109.e1–6. doi: 10.1016/j.urology.2012.12.027 23522297

[41] Chan TA Yarchoan M Jafee E Swanton C Quezada SA Stenzinger A. Development of Tumor Mutation Burden as an Immunotherapy Biomarker: Utility for the Oncology Clinic. Ann Oncol (2019) 30(1):44–56. doi: 10.1093/annonc/mdy495 30395155 PMC6336005

[42] Patel VG Oh WK Galsky MD. Treatment of Muscle-Invasive and Advanced Bladder Cancer in 2020. CA Cancer J Clin (2020) 70(5):404–23. doi: 10.3322/caac.21631 32767764

[43] Samstein RM Lee CH Shoushtari AN Hellman MD Shen R Yanjigian YY. Tumor Mutational Load Predicts Survival After Immunotherapy Across Multiple Cancer Types. Nat Genet (2019) 51(2):202–6. doi: 10.1038/s41588-018-0312-8 PMC6365097 30643254

[44] Chen L Liu S Tao Y. Regulating Tumor Suppressor Genes: Post-Translational Modifications. Signal Transduct Target Ther (2020) 5(1):90. doi: 10.1038/s41392-020-0196-9 32532965 PMC7293209

[45] Kacew A Sweis RF. FGFR3 Alterations in the Era of Immunotherapy for Urothelial Bladder Cancer. Front Immunol (2020) 11:575258. doi: 10.3389/fimmu.2020.575258 33224141 PMC7674585

[46] Chen DS Mellman I. Elements of Cancer Immunity and the Cancer-Immune Set Point. Nature (2017) 541(7637):321–30. doi: 10.1038/nature21349 28102259

[47] Licciardello MP Müllner MK Dürnberger G Kerzendorfer C Baidol B Trefzer C. NOTCH1 Activation in Breast Cancer Confers Sensitivity to Inhibition of Sumoylation. Oncogene (2015) 34(29):3780–90. doi: 10.1038/onc.2014.319 25263445

[48] Sharma P Callahan MK Bono P Kim J Spiliopoulou P Calvo E. Nivolumab Monotherapy in Recurrent Metastatic Urothelial Carcinoma (Checkmate 032): A Multicentre, Open-Label, Two-Stage, Multi-Arm, Phase 1/2 Trial. Lancet Oncol (2016) 17(11):1590–8. doi: 10.1016/S1470-2045(16)30496-X PMC5648054 27733243

[49] Balar AV Kamat AM Kulkarni GS Uchio EM Boormans JL Roumiguié M. Pembrolizumab Monotherapy for the Treatment of High-Risk non-Muscle-Invasive Bladder Cancer Unresponsive (KEYNOTE-057): An Open-Label, Single-Arm, Multicentre, Phase 2 Study. Lancet Oncol (2021) 22(7):919–30. doi: 10.1016/S1470-2045(21)00147-9 34051177

[50] Bellmunt J Paz-Ares L Cuello M Albiol S Guillem V Gallardo E. Gene Expression of ERCC1 as a Novel Prognostic Marker in Advanced Bladder Cancer Patients Receiving Cisplatin-Based Chemotherapy. Ann Oncol (2007) 18(3):522–8. doi: 10.1093/annonc/mdl435 17229776

[51] Gabrilovich DI. Myeloid-Derived Suppressor Cells. Cancer Immunol Res (2017) 5(1):3–8. doi: 10.1158/2326-6066.CIR-16-0297 28052991 PMC5426480

[52] Wing JB Tanaka A Sakaguchi S. Human FOXP3(+) Regulatory T Cell Heterogeneity and Function in Autoimmunity and Cancer. Immunity (2019) 50(2):302–16. doi: 10.1016/j.immuni.2019.01.020 30784578

[53] Yu X Lao Y Teng XL Li S Zhou Y Wang F. SENP3 Maintains the Stability and Function of Regulatory T Cells via BACH2 Desumoylation. Nat Commun (2018) 9(1):3157. doi: 10.1038/s41467-018-05676-6 30089837 PMC6082899

[54] Huang X Zuo Y Wang X Wu X Tan H Fan Q. SUMO-Specific Protease 1 Is Critical for Myeloid-Derived Suppressor Cell Development and Function. Cancer Res (2019) 79(15):3891–902. doi: 10.1158/0008-5472.CAN-18-3497 31186231

[55] Binnewies M Roberts EW Kersten K Chan V Gearon DF Merad M. Understanding the Tumor Immune Microenvironment (TIME) for Effective Therapy. Nat Med (2018) 24(5):541–50. doi: 10.1038/s41591-018-0014-x PMC5998822 29686425

[56] Lu JL Xia QD Lu YH Liu Z Zhou P Hu HL. Efficacy of Intravesical Therapies on the Prevention of Recurrence and Progression of non-Muscle-Invasive Bladder Cancer: A Systematic Review and Network Meta-Analysis. Cancer Med (2020) 9(21):7800–9. doi: 10.1002/cam4.3513 PMC7643689 33040478

