# Supplementary figures and images for: SUMOylation Pattern Predicts Prognosis and Indicates Tumor Microenvironment Infiltration Characterization in Bladder Cancer
Source: Front Immunol. 2022 Mar 28;13:864156. doi: 10.3389/fimmu.2022.864156 (PMC8995476; doi:10.3389/fimmu.2022.864156)

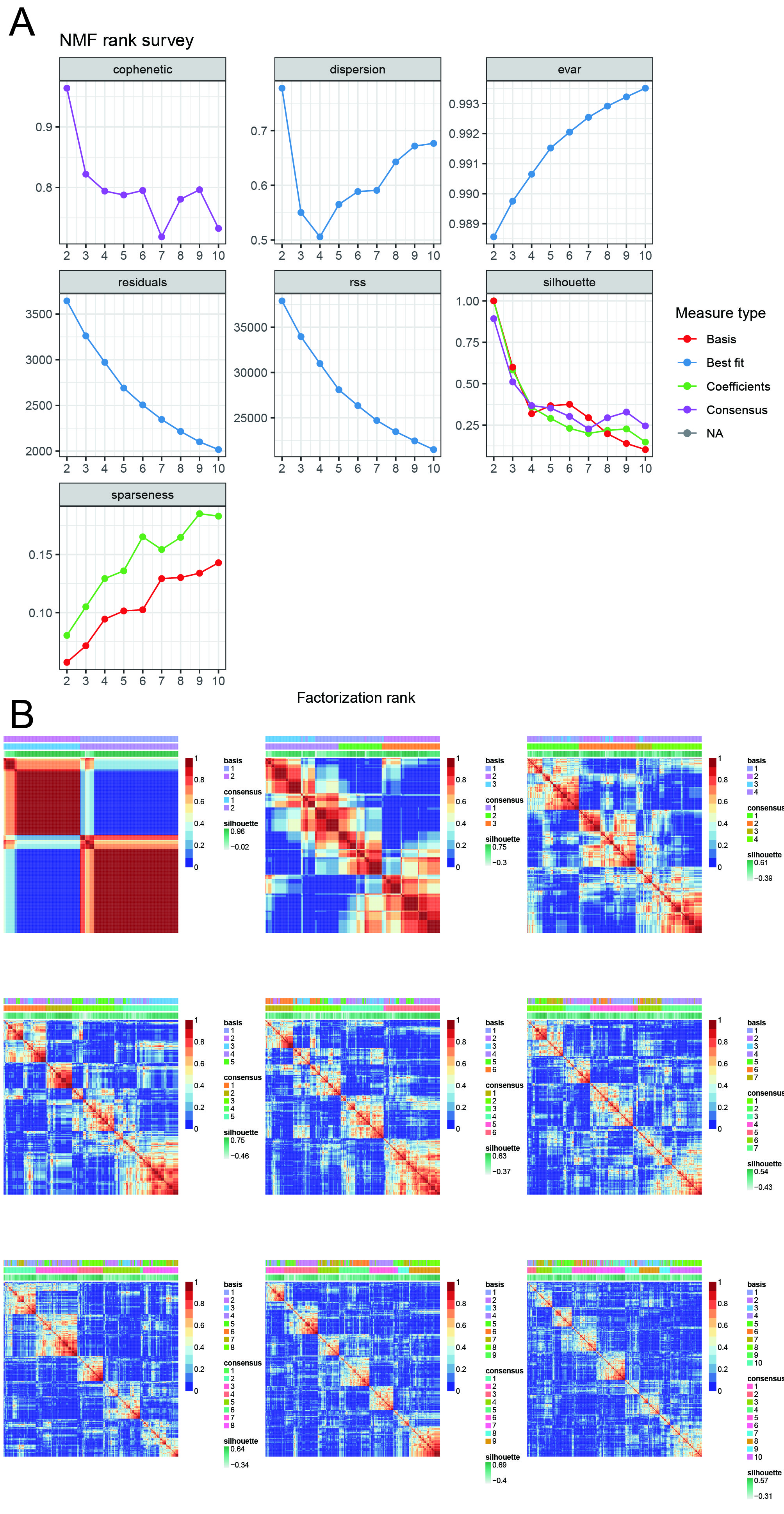

Supplement: Supplementary Figure 1 — The process of generating two distinct SUMOylation patterns using NMF. (A) When ranks from 2 to 10, there were changes in several cluster results related parameters such as the cophenetic correlation, residual sum of squares (RSS), and silhouette distribution et al. (B) Connectivity matrix for patients with bladder cancer in the meta-cohort by NMF when k ranged from 2 to 10. [file Image_1.jpeg]

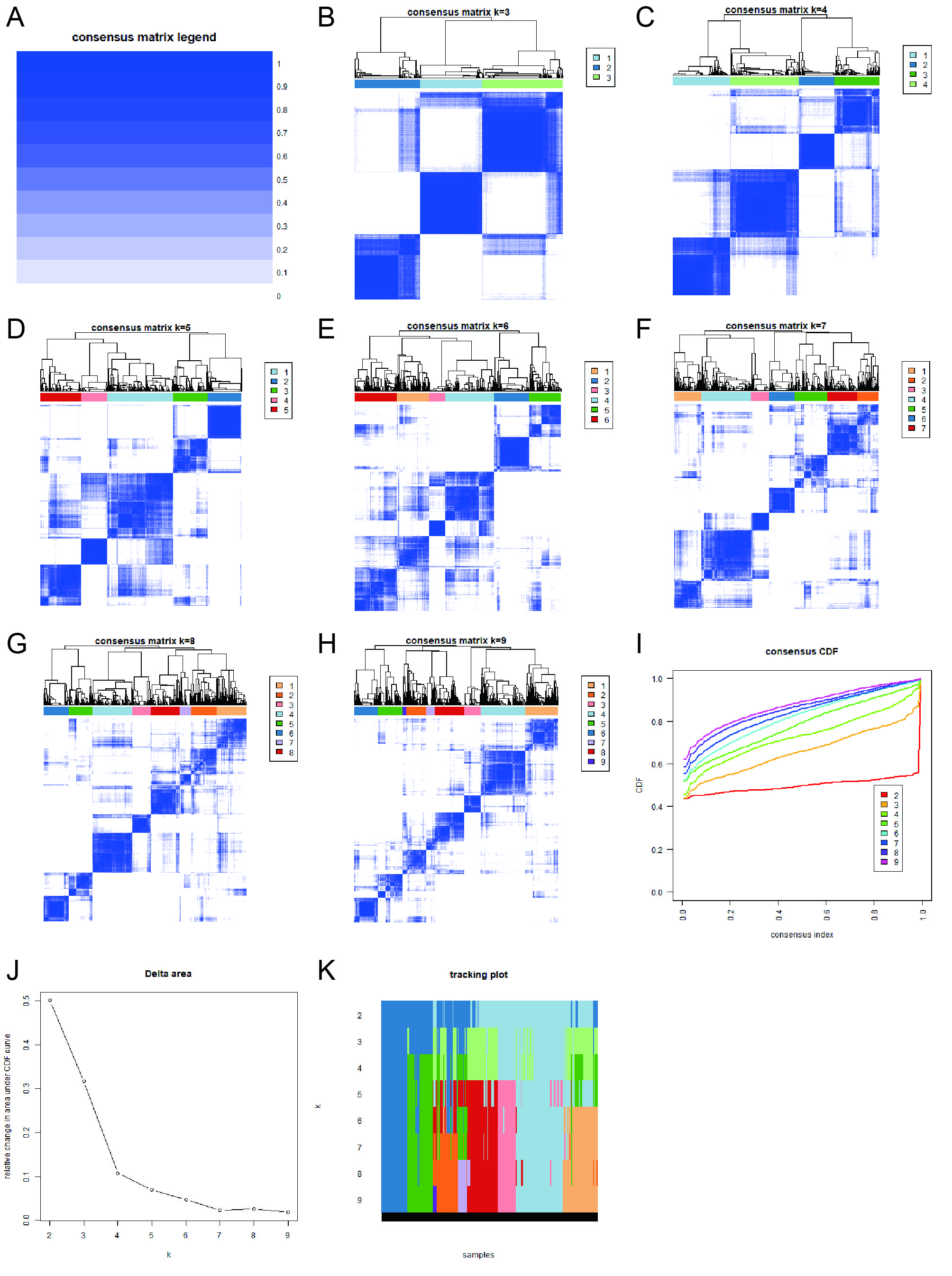

Supplement: Supplementary Figure 2 — The process of generating two distinct SUMO cluster related DEGs genomic patterns. (A–H) Unsupervised clustering of 1934 SUMO cluster related DEGs in meta-cohort and consensus matrices for k = 1, 3 - 9. (I) The cumulative distribution function (CDF) curve for k = 2 - 9. (J) The scree plot for k = 2 - 9. K The track plot for k = 2 - 9. [file Image_2.jpeg]

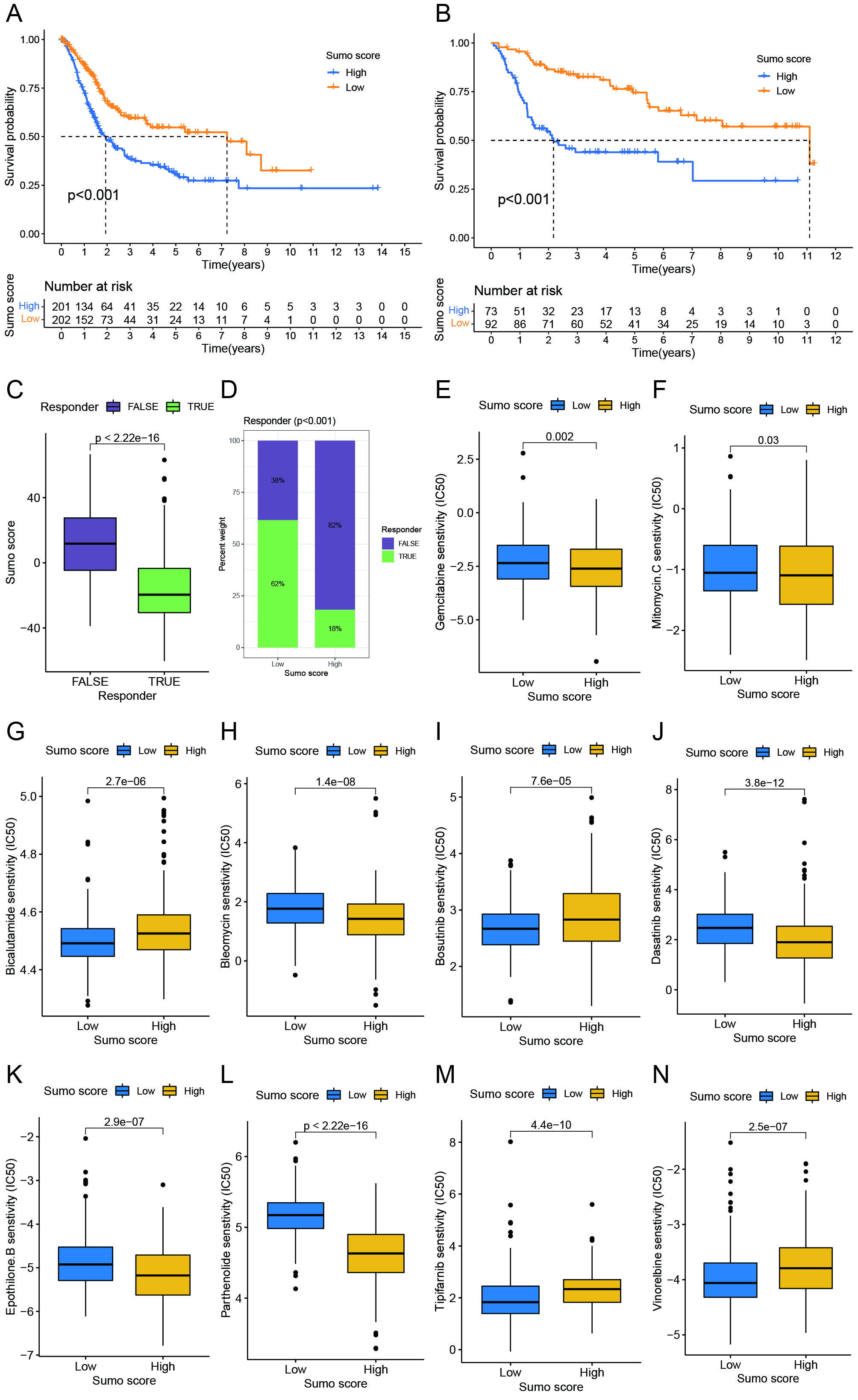

Supplement: Supplementary Figure 3 — The role of SUMOylation patterns in survival outcome and response to immunotherapy and chemotherapy. (A) Survival analyses for low (202 cases) and high (201 cases) SUMO score patient groups in TCGA-BLCA cohort using Kaplan-Meier curves (P < 0.001, Log-rank test). (B) Survival analyses for low (92 cases) and high (73 cases) SUMO score patient groups in GSE13507 cohort using Kaplan-Meier curves (P < 0.001, Log-rank test). (C) Differences in SUMO score between immunotherapy response and nonresponse groups in TCGA-BLCA cohort (P < 0.001, Wilcoxon test). (D) The proportion of patients who response to immunotherapy in low or high SUMO score groups. (E–N) Differences in IC50 of chemotherapy drugs between high and low SUMO score groups in meta-cohort. € Gemcitabine (P = 0.002, Wilcoxon test). (F) Mitomycin C (P = 0.03, Wilcoxon test). (G) Bicalutamide (P < 0.001, Wilcoxon test). (H) Bleomycin (P < 0.001, Wilcoxon test). (I) Bosutinib (P < 0.001, Wilcoxon test). (J) Dasatinib (P < 0.001, Wilcoxon test). (K) Epothilone (P < 0.001, Wilcoxon test). (L) Parthenolide (P < 0.001, Wilcoxon test). (M) Tipifarnib (P < 0.001, Wilcoxon test). (N) Vinorelbine (P < 0.001, Wilcoxon test). [file Image_3.jpeg]

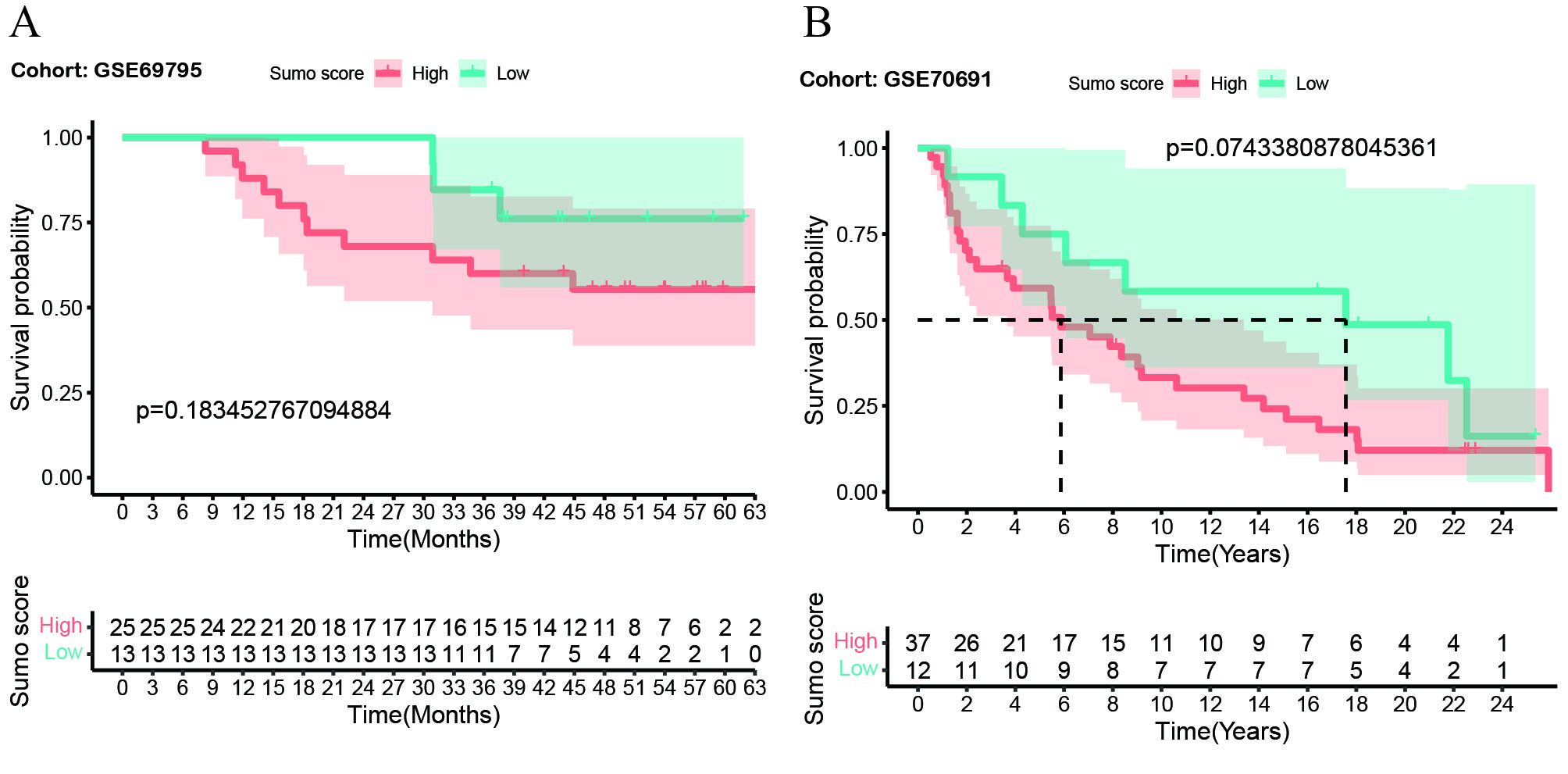

Supplement: Supplementary Figure 4 — Survival analyses for patients with high and low SUMO score in external validation dataset GSE69795 and GSE70691. (A) Survival analyses for low (13 cases) and high (25 cases) SUMO score patient groups in GSE69795 cohort using Kaplan-Meier curves (P = 0.183, Log-rank test). (B) Survival analyses for low (12 cases) and high (37 cases) SUMO score patient groups in GSE70691 cohort using Kaplan-Meier curves (P = 0.074, Log-rank test). [file Image_4.jpeg]

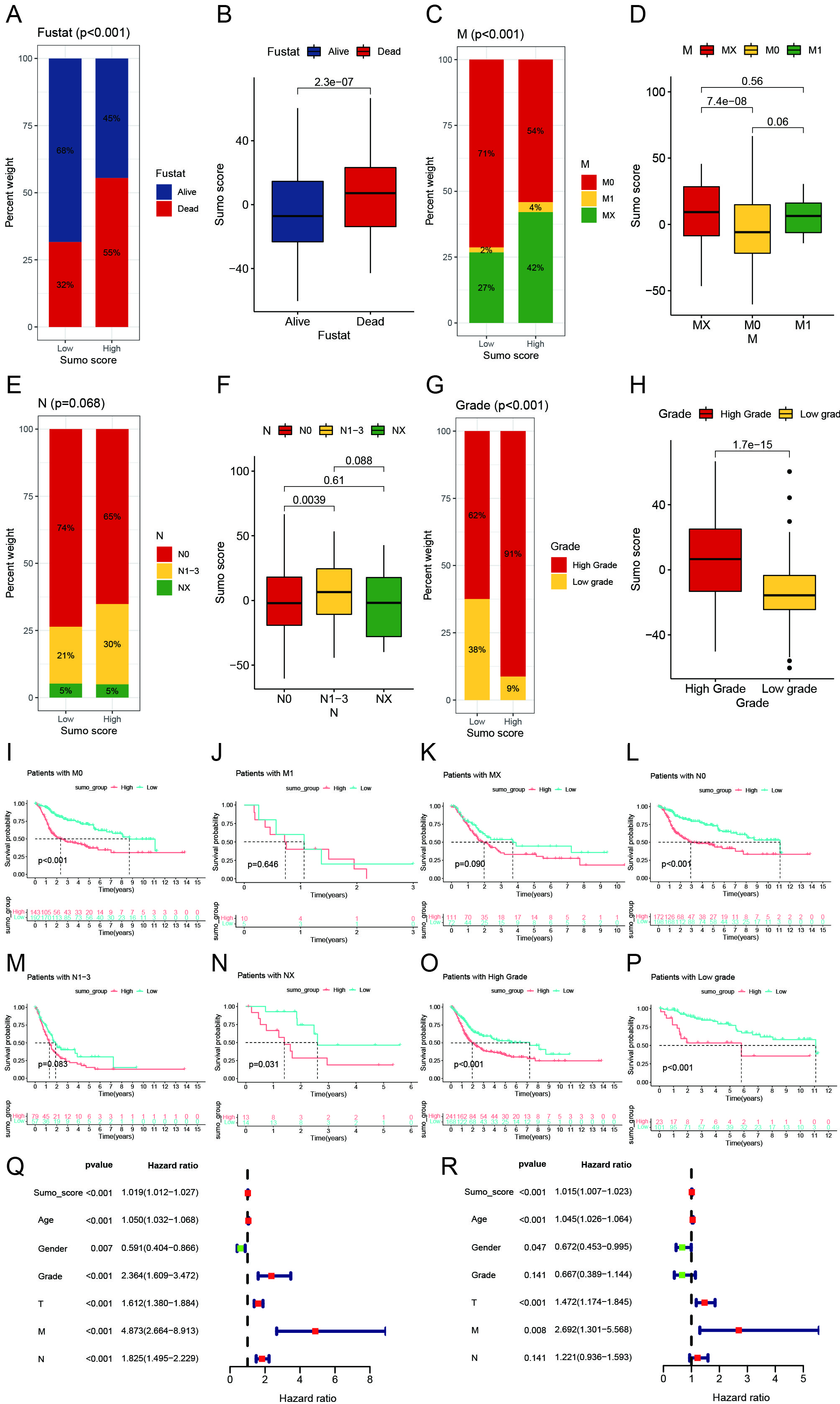

Supplement: Supplementary Figure 5 — Survival analyses for patients with high and low SUMO score in several subgroups. (A) The proportion of patients with different final survival status in low or high SUMO score groups. Alive/dead: 68%/32% in the low SUMO score groups and 45%/55% in the high SUMO score groups. (B) Differences in SUMO score between alive and dead subgroups in meta-cohort (P < 0.001, Wilcoxon test). (C) Differences in SUMO score among M0, M1 and MX groups in meta-cohort (Kruskal-Wallis H test). (D) The proportion of patients with M0, M1 and MX stage tumor in low or high SUMO score groups. € Differences in SUMO score among N0, N1-N3 and NX groups in meta-cohort (Kruskal-Wallis H test). (F) The proportion of patients with N0, N1-N3 and NX stage tumor in low or high SUMO score groups. (G) The proportion of patients with different tumor grade in low or high SUMO score groups. High grade/low grade: 62%/38% in the low SUMO score groups and 91%/9% in the high SUMO score groups. (H) Differences in SUMO score between high and low tumor grade groups in meta-cohort (P < 0.001, Wilcoxon test). (I-P) Survival analyses for high and low SUMO score groups in subgroups of meta-cohort using Kaplan-Meier curves. (I) Patients with M0 stage tumor subgroup (P < 0.001, Log-rank test). (J) Patients with M1 stage tumor subgroup (P = 0.646, Log-rank test). (K) Patients with MX stage tumor subgroup (P = 0.090, Log-rank test). (L) Patients with N0 stage tumor subgroup (P < 0.001, Log-rank test). (M) Patients with N1-3 stage tumor subgroup (P = 0.083, Log-rank test). (N) Patients with NX stage tumor subgroup (P = 0.031, Log-rank test). (O) Patients with high grade tumor subgroup (P < 0.001, Log-rank test). (P) Patients with low grade tumor subgroup (P < 0.001, Log-rank test). (Q) Univariate Cox regression analyses of OS in meta-cohort. The p-values were obtained by univariate Cox regression. (R) Multivariate Cox regression analyses of OS in meta-cohort. The p-values were obtained by multivariate Cox regression. [file Image_5.jpeg]
